# Deep learning enhanced light sheet fluorescence microscopy for in vivo 4D imaging of zebrafish heart beating

**DOI:** 10.1038/s41377-024-01710-z

**Published:** 2025-02-25

**Authors:** Meng Zhang, Renjian Li, Songnian Fu, Sunil Kumar, James Mcginty, Yuwen Qin, Lingling Chen

**Affiliations:** 1 School of Electronic and Information Engineering, Beihang University, Beijing, 100191 China; 2 College of Health Science and Environmental Engineering, Shenzhen Technology University, Shenzhen, 518118 China; 3 Institute of Advanced Photonics Technology, School of Information Engineering, Guangdong University of Technology, Guangzhou, 51006 China; 4 Photonics Group, Department of Physics, Imperial College London, London, SW7 2AZ UK

**Keywords:** Light-sheet microscopy, Biophotonics, Imaging and sensing

## Abstract

Time-resolved volumetric fluorescence imaging over an extended duration with high spatial/temporal resolution is a key driving force in biomedical research for investigating spatial-temporal dynamics at organism-level systems, yet it remains a major challenge due to the trade-off among imaging speed, light exposure, illumination power, and image quality. Here, we present a deep-learning enhanced light sheet fluorescence microscopy (LSFM) approach that addresses the restoration of rapid volumetric time-lapse imaging with less than 0.03% light exposure and 3.3% acquisition time compared to a typical standard acquisition. We demonstrate that the convolutional neural network (CNN)-transformer network developed here, namely U-net integrated transformer (UI-Trans), successfully achieves the mitigation of complex noise-scattering-coupled degradation and outperforms state-of-the-art deep learning networks, due to its capability of faithfully learning fine details while comprehending complex global features. With the fast generation of appropriate training data via flexible switching between confocal line-scanning LSFM (LS-LSFM) and conventional LSFM, this method achieves a three- to five-fold signal-to-noise ratio (SNR) improvement and ~1.8 times contrast improvement in ex vivo zebrafish heart imaging and long-term in vivo 4D (3D morphology + time) imaging of heartbeat dynamics at different developmental stages with ultra-economical acquisitions in terms of light dosage and acquisition time.

## Abstract (web summary)

The CNN-Transformer parallel network UI-Trans enables high-quality light sheet zebrafish heartbeat imaging, with ultra-economical acquisitions in terms of light dosage and acquisition time.

## Graphical abstract

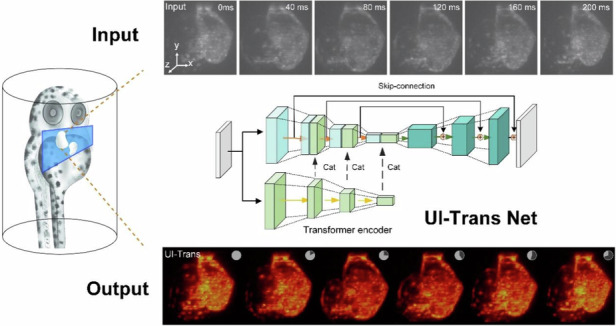

## Introduction

Fluorescence imaging technology is extremely powerful for revealing structural and functional information of specimens and has had a number of innovative advancements, including imaging strategies^1–3^ and labeling techniques^4–6^, offering exciting prospects for comprehensive studies of complex processes in many fields such as life science^7,8^, materials science, medicine and so on^9–14^. Specifically, there has been a rapid development of three-dimensional (3D) fluorescence imaging techniques in recent decades to provide molecular contrast with subcellular resolution throughout “mesoscopic” (millimeter-centimeter) samples due to the proliferating studies of biological processes at the whole-organism level^15^. Among those imaging techniques, light sheet fluorescence microscopy (LSFM) has gained increasing popularity due to its advantage of exceptional resolution and scale, low phototoxicity, and high speed, especially in the study of in vivo biological processes over an extended period of time (e.g. embryogenesis^16^, neuronal calcium dynamics^17^, etc.). Moreover, a number of sophisticated approaches in LSFM have been further developed to improve its performance and extend its applications. For instance, in terms of engineering the laser intensity profile, lattice light sheets provide improved axial resolution, rapid imaging with reduced phototoxicity/damage to the sample^18^, while Airy/Bessel beams, especially with two-photon excitation, provide a superior axial resolution across a wider lateral extent^19,20^. In terms of engineering the optical geometry, dual excitation/detection^21,22^, line confocal approaches^23–25^ and multi-modal integration OPTiSPIM^26^ can effectively reduce artifacts/blurring and alleviate the scattering problems associated with light sheet imaging. In terms of post-processing, deconvolution methods applied in LSFM with propagation-invariant beams^27,28^ can provide improved isotropic resolution and larger field of view.

While there are numerous improvement strategies of LSFM in various scenarios, its application to in vivo study of 3D cardiac dynamics of mesoscale biological samples (e.g. the model organism zebrafish) remains challenging, because imaging quality depends not only on the spatial resolution provided by the optical system, but also on the desired temporal resolution, the targeted fluorophore density and the total duration of a designed experiment as well as the inevitable scattering, photobleaching and phototoxicity of samples. Trade-offs are often made by considering the imaging speed, light exposure and resolution. In vivo imaging of beating zebrafish hearts typically requires an exposure time of less than 5 ms^29^, resulting in an extremely low signal-to-noise ratio (SNR) due to the restrictive maximal light exposure compatible with sample health. For the scattering issue, in vivo optical imaging is practical in some mesoscale organisms (e.g. *D. rerio* and *C. elegans)*, yet the image quality is deteriorated by the presence of scattering caused by refractive index inhomogeneity and morphological complexity of samples.

In order to address these challenges, denoising techniques^30,31^ have been widely employed in post-processing to recover meaningful information. Classical denoising methods, including spatial domain and transform domain methods (e.g. wavelet denoising, BM4D, non-local mean algorithms, etc^32–34^.), are regularly used for LSFM images. With the rapid advancement of deep learning, convolutional neural network (CNN) based methods (e.g. Noise2Noise, DeepCAD, residual channel attention network (RCAN), etc^35–37^.) have been developed in the field of digital image denoising and restoration, leading to superior performance compared to traditional algorithms^38^. Especially, there are a number of CNN-based networks specifically designed for denoising and restoring LSFM volumetric images, for the ease of visual analysis. A content-aware image restoration (CARE) network is able to address different kinds of image degradation such as low SNR due to low light dosages, axial resolution degradation, etc., by training with simulated images or actual high/low SNR images^39^. Isotropic divide stages-to-process double-ring modulation selective plane illumination microscopy (IDDR-SPIM) adopts a combination of DR-modulated light sheet and CNN to simultaneously achieve LSFM image denoising and volume imaging isotropy^40^. Complementary beam extraction combined the blind deconvolution and denoising (CBS-CBDD) achieves CNN network training for Bessel LSFM denoising and deconvolution^41^.

The above CNN-based methods have shown excellent capabilities in denoising, deconvolution and super-resolution applications, especially for microscopic images due to the readily accessible training data. However, mesoscopic imaging of in vivo zebrafish heart beating suffers from varying noise, inevitable uneven scattering, and complex moving biological structures^42^, all of which result in highly complex degradations in image quality. While these CNN-based methods hold the potential to enhance in vivo mesoscale-level volumetric imaging, the efficient strategy for ample training data acquisition, and the effectiveness for current methods to restore such coupled degradations remain to be explored, especially the latter one considering the challenge associated with the limited receptive field in these methods to address the scattered signals exhibiting extensive spatial variability over a relatively large spatial range and closely resembling the true signal in intensity.

Transformer, which is well-known for its ability in natural language processing, achieves superior performance in capturing intricate patterns and dependencies within sequential data through self-attention mechanisms^43^. Vision transformer (ViT)^44^, which has extended this transformative architecture to the realm of computer vision in recent years, have achieved remarkable results in various low-level tasks of biomedical image processing, e.g. image denoising, deblurring, and so on^45,46^. By combining transformer and CNN architectures, it becomes possible to comprehend complex scenarios while maintaining the sensitivity to local image details, thereby enhancing the network’s capacity to process intricate images^47^. There is an increasing interest in deep learning research to explore various combinations of Transformer and CNN architectures for diverse image processing applications, each offering different advantages on its designed purposes (details in Supplementary Table S1)^48–50^, yet more balanced hybrid architecture and refined training for addressing complex noise-scattering-coupled degradation remain to be explored. Furthermore, if confocal line-scanning LSFM (LS-LSFM)^23^ is integrated to generate high-quality scattering-alleviating ground truth (GT) datasets for training, the developed network is equipped with the ability to learn the mapping relationship from low-quality LSFM images to high-contrast GT, offering a new path in the restoration and enhancement of in vivo time-lapse volumetric LSFM imaging of mesoscale-level organisms.

Herein, we present an approach that addresses the aforementioned challenges in long-term in vivo mesoscale-level volumetric LSFM imaging of zebrafish heart beating. We develop a novel CNN-transformer network, namely U-net integrated transformer (UI-Trans), that successfully learns fine details while comprehending complex global features. With the fast generation of appropriate training data via easy switching between confocal LS-LSFM and conventional LSFM, this method enables high-quality image restoration in 3D mesoscopic fluorescence microscopy applications that always suffer from complex noise-scattering-coupled degradation. We characterize UI-Trans and other state-of-the-art networks (i.e. RCAN and CARE) and identify that UI-Trans provides superior performance in terms of denoising and scattering alleviation while better maintaining structural details/fidelity. Finally, we investigate the generalization capability of the pre-trained UI-Trans network and demonstrate three- to five-fold SNR improvement in multiple live samples, facilitating in vivo 4D high-quality imaging of zebrafish heartbeat dynamics at different developmental stages using time and light-efficient acquisitions.

## Results

### Developing Ul-Trans Network and the workflow for LSFM Enhancement

**Fig. 1:**
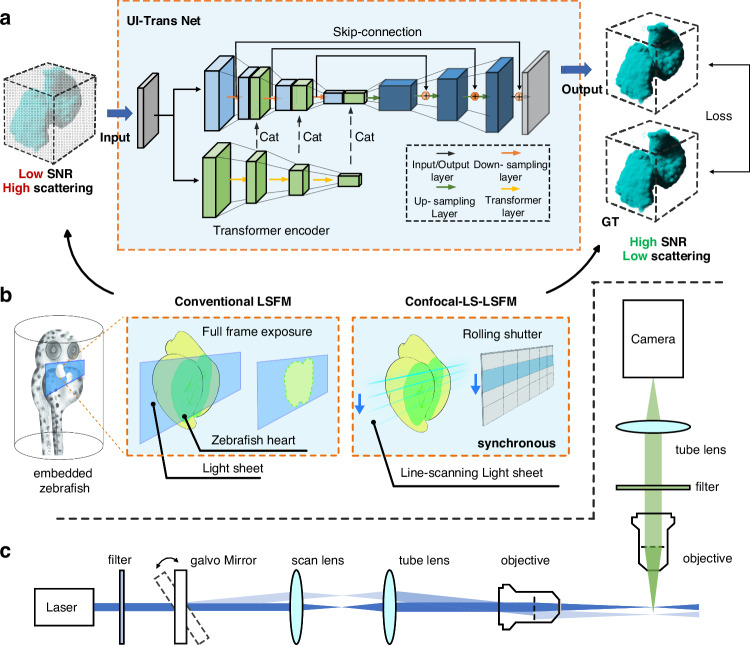
The architecture of UI-Trans network during the training phase in the context of the LSFM setup. **a** UI-Trans network is composed of a dual-branch encoder and decoder structure. The encoder consists of a convolutional encoder and a transformer encoder in parallel, repeatedly performing feature extraction and down-sampling. The decoder consists of alternating convolutional layers and up-sampling layers. **b** Low light-dosage LSFM images (using full-frame exposure) are used as input and the high light-dosage confocal LS-LSFM images (by synchronizing the light scanning and rolling shutter exposure) are used as the ground truth. **c** Optical geometry of LSFM system. Cat – concatenation

**Fig. 2:**
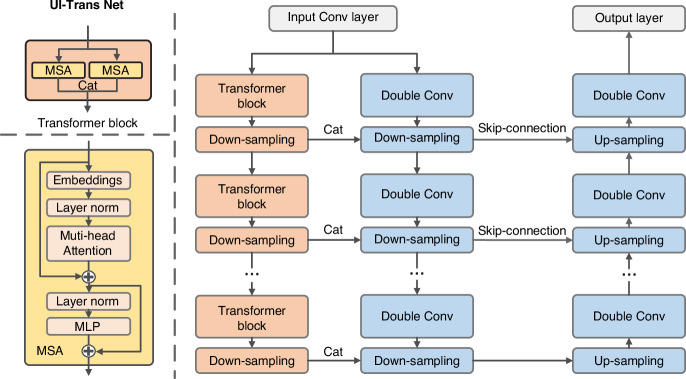
The architecture of UI-Trans network. Each transformer block consists of two parallel Multiheaded Self-Attention (MSA) modules. Each MSA module is constructed with an embedding layer, a layer norm layer, a multi-head attention layer, another layer norm layer and a multilayer perceptron layer. In the architecture of the transformer block, the channel dimension undergoes a doubling, whereas the dimension is subjected to a reduction by a factor of two through the process of down-sampling. Cat – concatenation, Conv – convolutional, MLP – multilayer perceptron

The architecture of our developed UI-Trans network in the context of the LSFM setup is illustrated in Fig. 1 and Fig. 2. As summarized in Fig. 1(a), a novel architecture was developed with a dual-branch encoder and decoder structure. In order to achieve better comprehension of complex global features while extracting details in feature maps, a transformer-based architecture is employed in one encoder branch and a multi-layer convolutional structure in the other. At each down-sampling stage, the image undergoes feature extraction and down-sampling in both the transformer and convolutional branches, with their output images concatenated along the channel dimension for the next stage. The decoder adopts the classic convolutional up sampling structure of U-Net. Before each up-sampling operation, skip connections are established at the corresponding positions in the encoder, enabling the integration of high-resolution features during the up-sampling process. More detailed network architecture information can be found in Materials and Methods B.

Figure 1b and c illustrates the optical geometry of the LSFM system using a virtual light sheet generated by a quick-sweeping Gaussian laser beam for the conventional LSFM mode. In contrast, for the confocal LS-LSFM mode, the rolling shutter exposure of the camera was switched on to synchronize with the scanning of the excitation beam, thereby reducing the contribution from scattered light and providing high-quality scattering-alleviated datasets as “ground truth” for training. With easy switching between conventional LSFM and confocal LS-LSFM, we achieve a readily accessible training dataset generation strategy, which enables pixel-to-pixel aligned image pair acquisition on the same imaging platform. A total of 400 paired images of fixed zebrafish heart, including low-quality LSFM images (with low exposure time and low illumination power) and high-quality confocal LS-LSFM images, as shown in Fig. 1b, were acquired as input and GT datasets, respectively. During the training, a multi-loss joint optimization was employed that fully considers the balance between pixel-level discrepancy (represented by mean absolute error (MAE)^51^) and perceptual image similarity (represented by perceptual loss computation using pre-trained VGG network^52^).

### 3D LSFM Image Denoising and Scattering-alleviating with Ul-Trans Net

#### Denoising

**Fig. 3:**
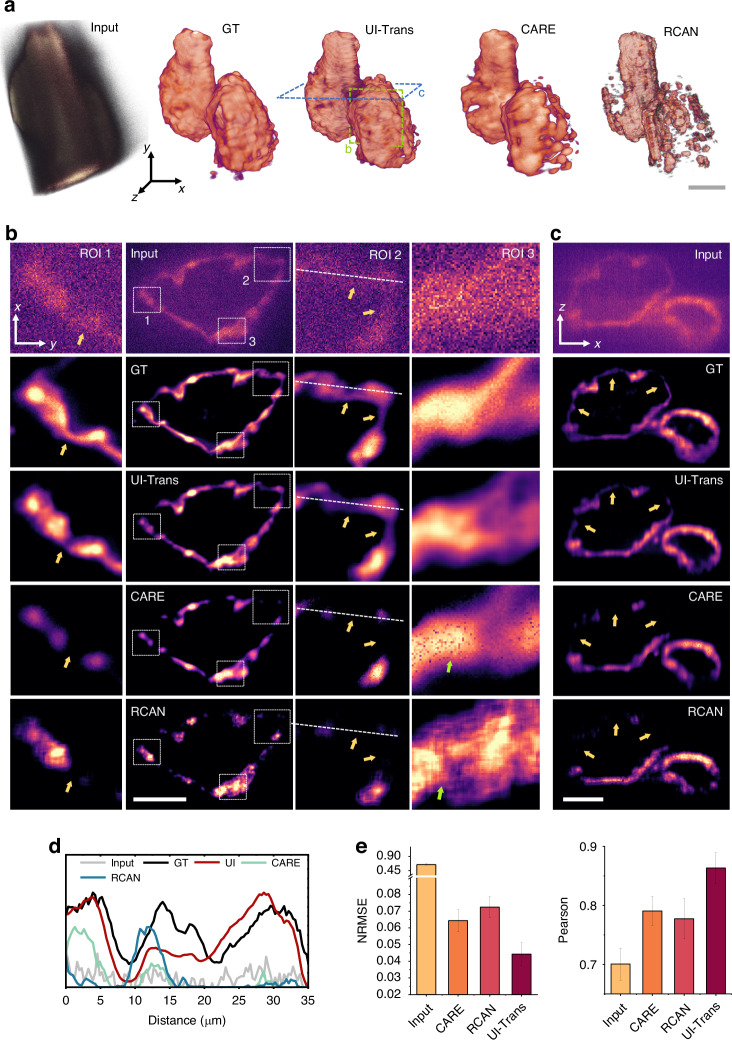
Ex vivo zebrafish heart volumetric imaging and the corresponding restorations using UI-Trans, CARE and RCAN. **a** 3D visualizations via surface blend method of a whole zebrafish heart acquired with low-quality LSFM (input), high-quality confocal LS-LSFM (GT) and the restored results using CARE, RCAN and UI-Trans. **b** 2D slice images in x-y plane and zoomed-in images in the ROI (white rectangle). **c** 2D slice images in x-z plane. **d** Cross-sectional intensity profile derived from the white lines in **b**. **e** NRMSE and Pearson coefficient calculated from the output restored images. Scale bars: 50 μm

In order to train the UI-Trans network, low-quality LSFM images of optically cleared zebrafish hearts were acquired by conventional LSFM using economical acquisitions (i.e. a low exposure time of 10 ms/plane and a low illumination power of 0.1 mW) with an average SNR of 6.2 dB (ranging from 4.5 -8.1 dB) as input. With the use of confocal LS-LSFM (using a high illumination power of 10 mW and an exposure time of 300 ms/plane), high-quality 3D images of a whole fixed zebrafish heart at 1 μm resolution with an average SNR of 15.1 dB (ranging from 12.5 - 20.4 dB) were obtained as GT data, but requiring 3000 times the light dosage and 30 times the acquisition time (the detailed characterization of SNR is in Supplementary Fig. S1). After the UI-Trans network is trained with multi-loss joint optimization, a higher SNR, more structural details and higher structural fidelity have been achieved in restorations on the validation set compared to other competitive networks, including CARE and RCAN. Figure 3a shows 3D visualizations of a whole zebrafish heart acquired with low-quality LSFM (input), high-quality LS-LSFM (GT), and the corresponding restored results using CARE, RCAN and UI-Trans network via surface blend method (more representation via maximum intensity projection (MIP) method and details can be found in Supplementary Fig. S2). Although the restored results from RCAN and CARE can partially discriminate signals from noise, they tend to lose some meaningful details and fail to match the visual quality of the GT (more detailed slice images in Supplementary Fig. S2). In order to quantify the extent of image improvement, normalized root mean square error (NRMSE)^53,54^ and Pearson coefficient^55^ of the output restored images using different networks were calculated and the results are shown in Fig. 3e, exhibiting ~31% / ~ 39% improvement of NRMSE and ~9% / ~ 11% higher performance of Pearson coefficient over CARE and RCAN respectively. (more comparison results on each individual data in the validation set can be found in Supplementary Fig. S3; other two commonly used evaluation metrics, i.e. peak signal-to-noise ratio (PSNR) and structural similarity index (SSIM), can also be found in Supplementary Fig. S4).

Moreover, in contrast to the simple image enhancement on signal strength from RCAN and CARE, the enhancement of UI-Trans network relies more on image features rather than local signal strength. The image restoration results of UI-Trans, RCAN, and CARE can be evaluated from 2D slice images. Figures 3b and c show slices of the 3D heart data represented in Fig. 3a (in the green and blue dashes boxes). UI-Trans network demonstrates an effective restoration of the slender myocardial layer (as shown in Figs. 3b and c), preserving intricate details that are lost in the output image of RCAN and CARE. By analyzing the zoomed-in slices of the atrial wall (Fig. 3b), it is also evident that UI-Trans network successfully reconstructs microscopic-level details with high similarity to the GT. In contrast, the images processed by RCAN and CARE exhibited a significant loss of weaker signals compared to UI-Trans, resulting in a discontinuous myocardial wall. In addition, when examining the cross-sectional intensity profiles (as shown in Fig. 3d) indicated by the dashed lines within the zoomed-in ROIs depicted in Fig. 3b, it is evident that both the peak position and curve shape indicate a higher degree of consistency between the image reconstructed by UI-Trans and the GT compared with the other networks. These results suggest that the enhancement of myocardial cells in zebrafish cannot be solely achieved by relying on the intensity of local signals. In the restored results, UI-Trans provides a clear visualization of myocardial cell distribution, whereas central myocardial cells in the output of RCAN and CARE remain unidentifiable. This advantage could be explained by the fact that the Transform block employed in the developed UI-Trans network can better leverage global information and then distinguish structural details from noise, thereby achieving higher visual quality compared to the other networks.

#### Scattering-alleviating

**Fig. 4:**
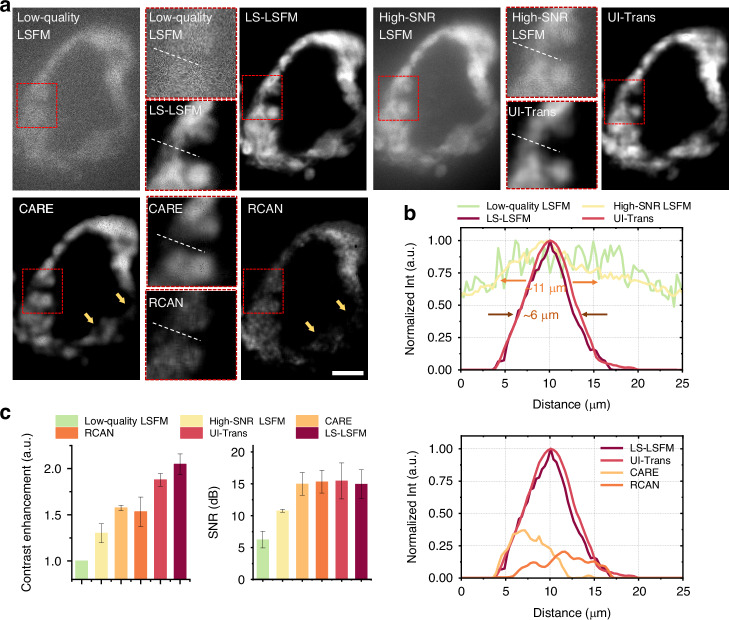
Scattering-alleviating investigation using ex vivo zebrafish heart volumetric imaging and the corresponding restored results. **a** 2D slice images acquired from a low-quality conventional LSFM (Input), high-SNR conventional LSFM, confocal LS-LSFM (GT), and the restored results from UI-Trans, CARE and RCAN, respectively. **b** Cross-sectional intensity profile derived from the white lines in **a**. **c** SNR and contrast enhancement calculated from the three kinds of acquired images and three kinds of the restored images. Scale bar: 20 μm

As aforementioned, the UI-Trans network achieved superior denoising performance while maintaining more structural details and higher fidelity, leading to perceptible differences in texture and accuracy. However, in vivo/ex vivo imaging of mesoscale organisms involves the inevitable phenomenon of scattering, which is not only sample-dependent but also varies with depth and illumination/detection direction, resulting in variable image degradation over the 3D image space. Although the SNR of acquired images can be enhanced by simply increasing the illumination power and exposure time of conventional LSFM, the additional imaging background/artefacts due to the uneven scattering cannot be addressed. In order to further illustrate the scattering-alleviating ability of the method developed via confocal LS-LSFM and UI-Trans network, high-SNR conventional LSFM images were acquired with an increased laser power of 1 mW and an extended exposure time of 500 ms/plane for comparison. Figure 4a depicts slice images of the fixed zebrafish heart acquired from low-quality conventional LSFM (Input to UI-Trans), high-SNR conventional LSFM, confocal LS-LSFM (GT), and the restored results from UI-Trans, CARE and RCAN, respectively. As demonstrated in previous studies, confocal LS-LSFM could effectively mitigate scattering that cannot be eliminated by simply increasing the illumination and exposure of conventional LSFM^25^, presenting improved contrast and a significant reduction in the scattered light haze. Importantly, the restored images enhanced by UI-Trans network closely resemble the GT images, and thus achieve superior performance in terms of scattering-alleviation. From the ROI area of the image (red boxes Fig. 4a), it is noted that even with 10 times laser power and 50 times acquisition time, the background caused by scattering still overwhelms the myocardium signal of high-SNR conventional LSFM, making it challenging to identify the edge. However, the ROI of the restored images enhanced by UI-Trans network exhibits clear myocardium edges. In order to further illustrate the differences, the cross-sectional intensity profiles were derived from dashed lines within the zoomed-in regions of ROIs (Fig. 4(b)), demonstrating that the restored results using UI-Trans (i.e. red line), which resembles the confocal LS-LSFM data (i.e. dark red line), can effectively remove the scattered light and restore the narrow structure of ~ 6 μm width, while the results from high-SNR LSFM image (i.e. yellow line) displays a clear background and ~ 11 μm width (i.e. 183% wider, showing blurring) compared to the confocal LS-LSFM ground truth.

In order to obtain an overall performance evaluation, quantitative analysis based on the calculation of SNR and contrast enhancement were employed and the comparison between different metrics is shown in Fig. 4c (more evaluation metrics such as Pearson coefficient and NRMSE in Supplementary Fig. S5). The results illustrate that UI-Trans network achieves a substantial improvement in SNR (~7.2 dB) and image contrast (~87%), in comparison with the limited effects on SNR (~4.5 dB) and image contrast (~30%) from the high-SNR LSFM acquisition. In addition, although RCAN- and CARE-restored results show comparable improvements in SNR (i.e. ~7.0 dB), they exhibit inferior improvements in contrast enhancement (i.e. ~57% and 53%, respectively) compared with UI-Trans. Since contrast enhancement is a key indicator of the improvement of the visually perceived contrast of images^23,56^, these results suggest there may be some difficulty for these networks in restoring the complex scattering-noise-coupled image degradations, even with the same training datasets. This could be attributed to the ability of UI-Trans to improve the global comprehension of the entire image space, thereby enhancing its performance in addressing complex noise-scattering-coupled degradation.

### In vivo Zebrafish Heartbeats Movie Restoration using pre-trained Ul-Trans Net

In vivo 3D mesoscopic imaging of zebrafish heart beat often requires extreme experimental conditions such as low illumination power and short exposure time for long-term live imaging considering the effects of phototoxicity and photobleaching, and thus results in low-quality images where cellular details and weak expression patterns are difficult to identify. In this section, in vivo imaging of zebrafish heartbeats was carried out using a low laser power of 0.1 mW and an extremely short exposure time of 3 ms/plane. This short exposure time was specifically required to suppress motion blur. We further characterized the generalization capability of the pre-trained UI-Trans network (i.e. exclusively on datasets from ex vivo acquisitions of fixed and optically cleared samples) for in vivo observations without using any additional ground truth data. It is noted that the simplest way to increase SNR is to increase laser power but this can temporarily stop the heart or even be detrimental to the sample (details in Movie S1).

#### In vivo zebrafish heart imaging enhancement with pre-trained networks

**Fig. 5:**
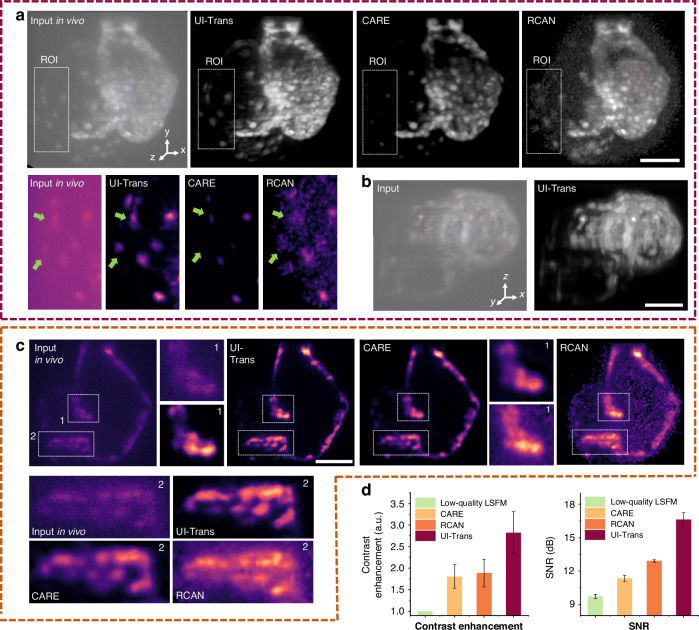
In vivo zebrafish heart volumetric imaging using deep-learning-enhanced LSFM. **a, b** 3D visualizations via MIP rendering of a whole zebrafish heart acquired with low-quality LSFM (input) and the corresponding restored results using pre-trained CARE, RCAN and UI-Trans respectively. **c** 2D slice images in *x-y* plane and the zoomed-in images of the ROI (orange rectangle). **d** Calculated SNR and contrast enhancement of the restored images. Scale bars: 50 μm

The heartbeat in a live 48 hours post fertilization (h.p.f.) transgenic zebrafish (Tg (myl7: GFP)) embryo was acquired and then image restoration was performed by the pre-trained network model including UI-Trans, CARE and RCAN. As the 3D visualizations of volumetric data via MIP rendering shown in Fig. 5a and b, it is difficult to identify myocardial cells in the raw data (Input) due to the spatiotemporally varying noise and uneven scattering. After restoration, it is noted that pre-trained UI-Trans network can better recover the details (e.g. atrium contour and myocardial cells as shown by green arrow) compared with CARE and RCAN, indicating better performance of UI-Trans. Figure 5c shows the 2D slice and the zoomed-in ROI images, exhibiting that UI-Trans-restored results can easily identify myocardial wall with more details and expression patterns (more 2D slice images at different spatial locations from the restored data and the elaborations in Supplementary Fig. S6 and Fig. S7).

In order to further quantify the enhancement results, the SNR and the contrast enhancement were calculated (Fig. 5d), showing a significant improvement of ~6.9 dB in SNR (i.e. increased from 9.71 dB to 16.63 dB) and a ~ 2.82 times contrast enhancement. It is also evident that the pre-trained UI-Trans network substantially outperforms the pre-trained CARE and RCAN, which achieve a ~ 1.6 dB/~3.2 dB improvement in SNR and a ~ 1.57 times/~1.49 times contrast enhancement, respectively. This performance of the pre-trained UI-Trans network is comparable to that achieved with the validation set, demonstrating the robustness and generalization ability of the developed method via confocal LS-LSFM and UI-Trans network model, despite the model having been trained on an ex vivo transparent heart dataset (more discussion in Supplementary Fig. S8).

#### In vivo cardiac dynamics movie enhancement by UI-Trans

**Fig. 6:**
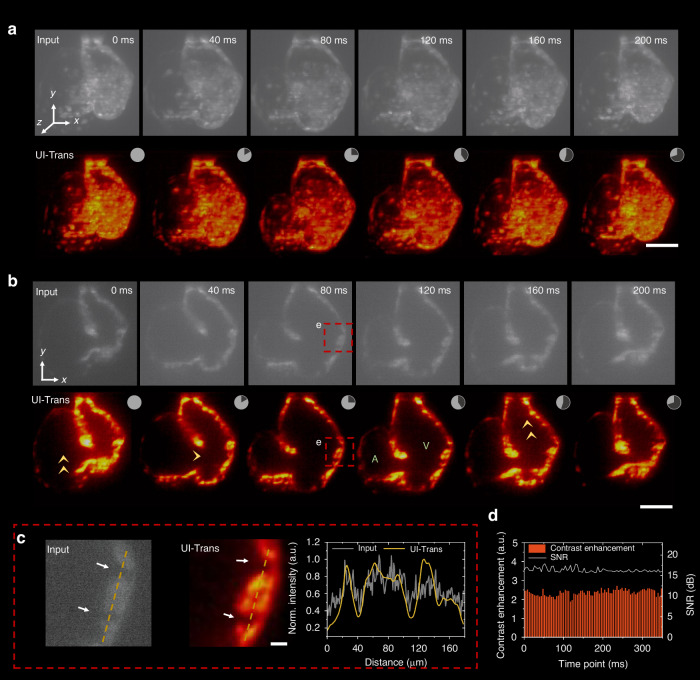
In vivo cardiac dynamics investigation using UI-Trans-enhanced LSFM imaging. **a** 3D visualizations via MIP rendering of the heartbeat enhanced by UI-Trans network at different time points within the same heartbeat cycle. **b** 2D slice images in *x-y* plane. **c** Zoomed-in images of the ROI (red rectangle) and the cross-sectional intensity profile along yellow dotted line. **d** Calculated SNR and contrast enhancement of images enhanced by UI-Trans at different time points within a heartbeat cycle. A, atrium; V, ventricle. Scale bars: 50 μm in **a** and **b**, 10 μm in **c**

In order to further illustrate the 4D in vivo imaging capability of the developed method via conventional LSFM and pre-trained UI-trans model, we extended the demonstration to study the morphology of a beating heart at video rate. Figure 6a and b show 3D visualizations via MIP rendering and 2D slice images of a heart enhanced by the pre-trained UI-Trans network at multiple time points within the same heartbeat cycle, demonstrating that the UI-Trans-restored images show comparable improvements in image quality to the validation set, including improved visibility of structural details compared to the input image (e.g. the ventricular characteristic trabeculae as shown in Fig. 6c). We note that applications of deep learning to fluorescence microscopy typically make time-independent predictions and do not capture temporal variations very well. The whole restored cardiac dynamics movie is shown in Movie S2, visually showing that the pre-trained UI-Trans network can provide high temporal consistency. The quantitative metrics (i.e. SNR and contrast enhancement) are shown over the duration of a single heartbeat cycle in Fig. 6d, demonstrating the temporally consistent enhancement results and further validating the robustness and stability of UI-Trans.

The improvements in clarity and contrast enable a more detailed observation of dynamic processes during the zebrafish heartbeat cycle. The start time of atrial diastole was defined as the initial time point, and the atrium reached its maximum dilation with the blood pumped into atrium (yellow arrow in Fig. 6b) at 40 ms and concurrently, the ventricle initiated its dilation. At 80 ms, the ventricle reached its maximum dilation while the atrium underwent compression, actively pumping blood from the atrium to the ventricle. Subsequently, the ventricle contracted, propelling blood into the bulbus arteriosus, while the atrium began to recover and expanded again, facilitating the influx of venous blood into the atrium. The overall duration of a heartbeat cycle was approximately 300 ms, and the observation of the dynamic heartbeat cycle was consistent with previous studies in terms of morphology and cardiac dynamics^57,58^.

#### In vivo cardiac dynamics movie enhancement at different developmental stages

**Fig. 7:**
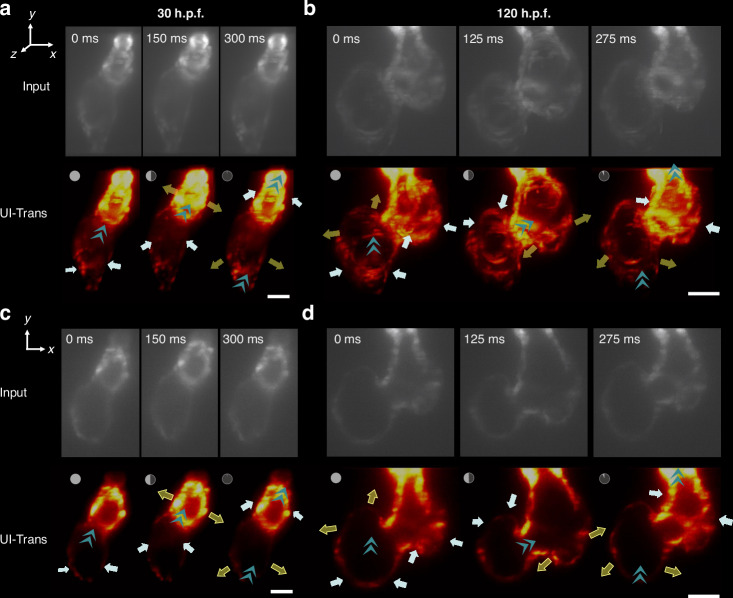
In vivo cardiac dynamics investigation at different developmental stages using UI-Trans-enhanced LSFM imaging. The enhanced cardiac dynamics 3D visualizations via MIP rendering of (**a**) embryonic stage (30 h.p.f.) and (**b**) late cardiac development (120 h.p.f.) with white and yellow arrows showing the contraction and relaxation of ventricles and atria while cyan arrowheads indicating the path of blood flow. **c**, **d** The corresponding 2D slice images of (**a**) and (**b**) in *x-y* plane. Scale bars: 50 μm

The morphology of zebrafish hearts varies significantly at different developmental stages. In order to further evaluate the general applicability of UI-Trans for different samples, we extended the demonstration of in vivo 4D imaging of zebrafish heartbeats to some different developmental stages. Specifically, the in vivo cardiac dynamics movie at the embryonic stage (i.e. 30 h.p.f.) and at late cardiac development (i.e. 120 h.p.f.) were acquired and enhanced as described previously. Figure 7 shows the 3D visualizations via MIP rendering and 2D images of the heart captured at different timepoints during the heartbeat cycle and their corresponding restored results, exhibiting the robustness of pre-trained UI-Trans network in processing LSFM images of varying cardiac shapes in different developmental stages. After UI-Trans enhancement was applied, the ventricle can be clearly separated from the background, which was difficult to discern in the input image. The SNR of the image has been significantly improved, i.e. increasing from 9.27 dB to 14.30 dB and from 9.58 dB to 13.69 dB for 30 h.p.f. and 120 h.p.f. zebrafish respectively, providing visually-pleasing heartbeat movies (Movie S3 and Movie S4) to follow the characteristic structures of the heart throughout the cardiac cycle at various developmental stages. For example, it is observed that the heartbeat process manifests as contractile waves in 30 h.p.f. embryos (Fig. 7a and c), while the zebrafish heart in late cardiac development (i.e. 120 h.p.f.), with complete differentiation of the atrium and ventricle, exhibits a more intricate heartbeat process (Fig. 7b and d). Moreover, it is noted that zebrafish embryos of 30 h.p.f. have not yet hatched, and there is no such early heart imaging data in the training set. This further demonstrates the powerful generalization applicability of the developed UI-Trans network. These results demonstrate that the developed method can provide high-speed high-quality 4D imaging of intact beating zebrafish hearts at different developmental stages, facilitating new insights into cardiac studies without fixation artifacts.

## Discussion

The results demonstrate that the combination of confocal LS-LSFM and the UI-Trans network not only achieves superior denoising performance while maintaining structural details/fidelity, but also alleviates uneven scattering, both of which are extremely useful for in vivo mesoscopic imaging over extended periods of time. The functionality of key components of the UI-Trans network was analyzed through ablation experiments, demonstrating the efficacy of combining both vision transformer and basic U-type CNN architectures to provide a comprehensive view of both local and global image features as well as an enhanced feature extraction capability (ablation results and detailed explanation in Supplementary Fig. S9 and Table S2). This combination also brings challenges, including the balance between denoising performance and fidelity maintaining, and the increased model complexity. To achieve a satisfied trade-off between noise reduction and detail preservation, a multi-loss joint optimization was employed to consider the balance between pixel-level discrepancy and perceptual image similarity, and the effects of this optimization strategy were also analyzed through ablation experiments (results in Supplementary Fig. S9 and Table S2). In addition, the effects of the amount of training data on denoising and detail preservation performance were analyzed, and the results demonstrate that a significant enhancement in image quality can be achieved with a modest dataset of 20 and 50 samples. However, further increases in the training data can lead to even higher performance (more details in Supplementary Fig. S10). While the developed UI-Trans demonstrates the excellent capabilities in addressing the complex image degradation problem of miscellaneous varying noise and uneven scattering, this success critically depends on proper adaptation to the task specifics, efficient handling of the model complexity, a sufficient amount of high-quality training data, and ensuring that the model do not overfit to particular noise types or training scenarios. It should also be noted that UI-Trans is not appropriate for intensity-based quantification applications considering the nonlinear nature of its predictions and its training process.

Moreover, classical algorithms (including BM4D and non-local means (NLM)) were used for the enhancement of low-quality LSFM images and the results were also compared with deep learning methods (Fig. S11), showing that deep learning models significantly outperformed classical algorithms in both visualization and quantitative metrics on the datasets in this study. However, classical algorithms retain a number of advantages, including consistency between input and output and adaptability across diverse categories of images. Furthermore, we evaluate experimental settings in terms of imaging performance for different methods (i.e. low-quality conventional LSFM, high-SNR conventional LSFM, confocal LS-LSFM and three deep-learning-enhanced LSFM modalities) in terms of illumination power, acquisition time, total light exposure, SNR and contrast enhancement. As shown in Fig. S12, UI-Trans-enhanced LSFM achieves competitive high-quality images with confocal LS-LSFM but requiring less than 0.03% of the light exposure and 3.3% of the acquisition time. This reduction in light exposure and acquisition time is particularly crucial for long-term live imaging of fast processes, since it not only effectively minimizes the adverse effects of prolonged light exposure on the subject organisms but also facilitates rapid imaging with low-intensity light.

Another key feature of our approach is the rapid and straightforward generation of appropriate training data via the flexible switching between conventional LSFM and confocal LS-LSFM on the same imaging system. It not only provides high-quality, scattering-suppressed ground truth data rather than the conventional high-SNR data by simply increasing light dosage, but also enables pixel-to-pixel aligned image pair acquisition which reduces additional labors for image registration and reduces the training difficulty. Via the developed UI-Trans network and confocal LS-LSFM, this approach successfully addresses the complex image degradation problem of miscellaneous varying noise and uneven scattering, allowing the photon budget savings during imaging to improve the acquisition parameters for different application scenarios, e.g. imaging speed, experimental duration, light power, phototoxicity and photobleaching. The generalization capability of the pre-trained UI-Trans network shows three- to five-fold SNR improvement in multiple live samples, facilitating in vivo 4D high-quality imaging of zebrafish heartbeat dynamics at different developmental stages with economical acquisitions in terms of light dosage and acquisition time (more exploration on further analysis in Supplementary Fig. S13). While the UI-Trans network is trained for a specific organism and fluorescent markers, it is indeed one of supervised deep-learning models that can automatically learn the mapping between input data and desired output when given ample and appropriate training data. To explore the applicability of the developed UI-Trans for other organisms, the microscopy recordings of developing Tribolium castaneum embryos, *S. mediterranea* flatworm and *D. melanogaste*r drosophila wing (i.e. online accessible from CARE datasets^39^) were used as extra samples, and the restored results demonstrate that UI-Trans can perform well on a wide range of applications with proper adaptation, especially for more complex degradation tasks (details in Supplementary Fig. S14 and Fig. S15). Future explorations of joint-training with different microscopy techniques on various organisms will further broaden the applicability of UI-Trans to address more complex biological imaging tasks. We believe that the method developed here can be also adapted to a wider range of image types and will open new paths for high-quality time-resolved volumetric imaging to investigate spatial-temporal dynamics of organism systems.

## Materials and Methods

### LSFM Setup

Figure 1c illustrates the optical geometry of the LSFM system. In the excitation path, the laser beam was generated by a semiconductor laser (Colbolt, 473 nm) and then magnified 5× by two lenses (AC254-150-A-ML, AC254-30-A-ML, Thorlabs). Subsequently, the beam was scanned using a digital scanning system (i.e. a galvanometer (GVS212, Thorlabs) with associated scan lens (GAS0121, Thorlabs)) and then focused by an illumination objective lens (LMPlanFLN 10×, NA 0.25, Olympus) on the center of the sample from one side. In the detection path (orthogonal to the illumination path), the fluorescence distribution of the sample was captured by using a sCMOS camera (Prime BSI, Photometric) coupled with a detection objective lens (LUCPlanFLN 20×, NA 0.45, Olympus) and a corresponding tube lens (TTL180-A, Thorlabs). The sample was moved by a PZT motor (Coretomorror, P73Z) along the z-axis, enabling full-depth imaging. In order to operate under conventional LSFM mode, the galvanometer in the illumination path swept quickly at 106 Hz and generated a digital virtual light sheet, providing a lateral/axial resolution of 0.98 μm/4 μm at an acquisition time of 10 ms for each plane. In contrast, for the confocal LS-LSFM mode (i.e. to generate a scattering-alleviating dataset), the rolling shutter exposure of the camera was synchronized with the scanning of the excitation beam in the y-direction, thereby improving rejection of scattered light and providing higher-contrast images with the same resolution but at an acquisition time ~300 ms for each plane. All the acquisition settings and hardware control used an in-house developed LabVIEW program.

### UI-Trans Net Model

We developed the UI-Trans network by employing a hierarchical encoder-decoder architecture as shown in Fig. 2. To enhance both long-range attention and the restoration of details, a novel encoder architecture was proposed based on self-attention transformer and convolution collaborative feature extraction. Each branch is composed of 3 alternating feature extraction modules (Transformer block or double convolution block) and down-sampling layers. Within the transformer block, two Multiheaded Self-Attention (MSA) modules are employed and their outputs are concatenated to align with the channel dimensions of the output produced by the double convolution block. The output of each transformer block, following down-sampling, is concatenated with the down-sampled output of the corresponding convolutional layer as supplementary features. The decoder pathway is symmetrical to the convolutional branches in the encoder pathway, employing a combination of double convolutional layers and up-sampling alternately to restore the image size and details. Here, skip connections are established at the corresponding level of encoder pathway to transfer fine-grained information. (More ablation experiment results in Fig. S9 and Table. S2).

The MSA module is used to capture global features. Firstly, the embedding layer transforms the input three-dimensional image into a sequence. Given that the transformer module is primarily designed to concentrate on the global features of the image, an interval extraction method is adopted in the embedding layer to obtain patches^59^. Subsequently, the sequence is processed through layer normalization, self-attention, another layer normalization and a multilayer perceptron (MLP), providing 3D image output with the same size of the original input. Two residual connections are included to prevent gradient vanishing.

### Sample Preparation

Optically cleared transgenic zebrafish (Tg (myl7: GFP), China Zebrafish Resource Center) were used as examples to acquire input-GT datasets. A total of 100 embryos aged 24–120 hours post-fertilization (h.p.f.) were euthanized by immersing in a 2500 mg/L MS-222 (tricaine, Sigma A5040) solution for 20 minutes^60^. Then, they were immersed in paraformaldehyde (PFA) for approximately 30 minutes and washed 3-5 times with phosphate-buffered saline (PBS). Afterwards, they were optically cleared using the UbasM method^61^ by immersing the samples in UbasM1 solution for 30 minutes, washing with PBS, and then embedding in a cylinder of 1.5% w/w agar (Agar, Sigma A1296) and were then immersed in UbasM2 solution for 3 days. The embedded zebrafish embryos were finally mounted on the motor stage and subsequently imaged in UbasM2 solution.

For in vivo LSFM imaging of zebrafish heartbeats, the zebrafish embryos were anesthetized with 150 mg/L of tricaine for 20 minutes and then embedded in 1.5% agar cylinders. The agar block was then fixed on the motor stage and immersed in water for imaging.

### Zebrafish Heart Datasets Acquisition and Preprocessing

In order to train the UI-Trans network, low-quality LSFM images (with a laser power of 0.1 mW and an exposure time of 10 ms for a plane) and high-quality confocal LS-LSFM images of optically cleared zebrafish hearts (with a laser power of 10 mW and an exposure time of 1 ms for each line, i.e. resulting an exposure time of 300 ms per plane) were acquired as input and GT datasets. In order to confirm alignment between input and GT images at pixel level, the images were aligned through rigid translation by calculating the Pearson correlation coefficient in all 3 directions. Subsequently, the images were cropped to a size of 256 × 256 × 256 for training (0.325 μm per voxel).

### In vivo Zebrafish Heartbeat Imaging and Reconstruction

In vivo 4D (i.e. 3D spatial + time) zebrafish heartbeat imaging was acquired using conventional LSFM with an exposure time of 3 ms/plane and a laser power of 0.1 mW/plane. For each slice, 1000 consecutive time series images were collected, and the heartbeat 4D image was reconstructed by applying a synchronization method to the movie stacks post-acquisition^29^ (more details in Fig. S16).

### Training

The UI-Trans network was optimized using the ADAM optimizer^62^ with the deep learning framework PyTorch 2.00 on a Dell T7920 workstation with two NVIDIA A6000 GPUs, two Intel Xeon Gold 6240 CPUs of 2.60 GHz and 384 GB RAM. A multi-loss joint optimization strategy was employed that fully considers the balance between pixel-level discrepancy and perceptual image similarity. The mixing training loss was formulated as a weighted sum of *L*_*MAE*_ and *L*_*vgg*_ as

$$\begin{array}{l}{L}_{{\rm{total}}}={L}_{MAE}+\lambda {L}_{{\rm{vgg}}}=\frac{1}{D\times H\times W}{\sum }_{D,H,W}|{V}_{D,H,W}\\\qquad\qquad\qquad\qquad\qquad\quad-\,{\hat{V}}_{D,H,W}|+\lambda {\sum }_{x}{L}_{vgg-2d}({S}_{x},{G}_{x})\end{array}$$

where *L*_*MAE*_ represents the pixel-level mean absolute error (MAE)^51^, *L*_*vgg*_ represents the perception loss of the output and the GT^52^, *V* is the restored image output and $\hat{V}$ is the GT image. *D, H* and *W* represent the depth, the height and the width of the image respectively. By appropriately setting the weighting parameter *λ*, the joint loss could balance the preservation of pixel-level similarity and the extraction of global structure similarity. Considering the order of each loss and the required balance in this study, *λ* was set to 0.01. Given that the pre-trained VGG network used for perceptual loss computation was originally designed for 2D images, 3D acquired images were divided into 2D slices and the average perceptual loss was calculated subsequently on a per-volume basis. A total of 400 pairs of ‘input-GT’ images of zebrafish hearts were acquired. The validation set and training set were randomly sampled from the isolated cardiac dataset in the ratio of 9:1.

### Evaluation

In order to implement an overall evaluation, both direct visualization of the UI-Trans enhanced images and six quantitative metrics were employed in this study, including normalized root mean square error (NRMSE)^53,54^ (small values indicating better performance), Pearson coefficient^55^, peak signal-to-noise ratio (PSNR), structural similarity index (SSIM)^63^, SNR^40^, and contrast enhancement^23,56^ (large values indicating better performance). UI-Trans is compared with two competitive deep learning methods, i.e. CARE using the popular U-net neural network architecture with minor modifications at the output layer^39^ and RCAN employing multiple skip connections between network layers as well as a channel attention mechanism^37^. Both methods were trained by the use of open-source code using the parameters recommended in the corresponding studies.

Image processing was performed using MATLAB 2022b and 3D reconstruction/renderings performed using Imaris 9.0.1 software. For direct visualization comparison, the display intensity of the images presented have been individually adjusted for the individual optical contrast.

## Supplementary information

Supplementary Information

movie S1

movie S2

movie S3

movie S4

## Data Availability

All the data that support the findings of this study are available from the corresponding author on request.
