# Supplementary material for: Deep learning enhanced light sheet fluorescence microscopy for in vivo 4D imaging of zebrafish heart beating
Source: Light Sci Appl. 2025 Feb 25;14:92. doi: 10.1038/s41377-024-01710-z (PMC11850918; doi:10.1038/s41377-024-01710-z)
Supplement: Supplementary file 1 — Supplementary Information [file 41377_2024_1710_MOESM1_ESM.docx]

**Supplementary Information for**

**Deep learning enhanced light sheet fluorescence microscopy for *in vivo* 4D imaging of zebrafish heart beating**

**Meng Zhang,^1^ Renjian Li,^1,2^ Songnian Fu,^3^ Sunil Kumar, ^4^ James McGinty,^4^ Yuwen Qin,^3,*^ Lingling Chen^2,*^**

*^1^School of Electronic and Information Engineering, Beihang University, Beijing, 100191, China*

*^2^College of Health Science and Environmental Engineering, Shenzhen Technology University, Shenzhen, 518118, China*

*^3^Institute of Advanced Photonics Technology, School of Information Engineering, Guangdong University of Technology, Guangzhou, 51006, China.*

*^4^Photonics Group, Department of Physics, Imperial College London, London, SW7 2AZ, UK*

**chenlingling@sztu.edu.cn*

[**qinyw@gdut.edu.cn*](mailto:*qinyw@gdut.edu.cn)

**This Word file includes:**

Table S1. CNNs with transformer on low-level CV tasks.

Fig. S1. SNR characterization of the developed imaging system.

Fig. S2. Restored results using CARE, RCAN and UI-Trans network via maximum intensity projection (MIP) method.

Fig. S3 and S4. Quantitative analysis based on evaluation metrics on each individual data in the validation set.

Fig. S5. Scattering-alleviating investigation using 2D image comparisons.

Fig. S6. 2D slice images of Input and UI-Trans-restored data of in vivo heartbeat imaging.

Fig. S7. 2D slice images of Input and restored data using pre-trained UI-Trans, CARE and RCAN for *in vivo* heartbeat imaging.

Fig. S8. The Input, GT and UI-Trans-restored images at relatively higher SNR level in *ex vivo* datasets.

Fig. S9. The input and output images of the networks in the ablation experiment.

Table. S2. The ablation experiment results.

Fig. S10. Performance evaluation of UI-Trans using a reduced image set for training.

Fig. S11. Restoration results using classical algorithms.

Fig. S12. The trade-offs of LSFM imaging between imaging speed, light exposure and image quality.

Fig. S13. Exploration on further analysis for heartbeat rhythm information.

Fig. S14 and S15. Applicability exploration of UI-Trans on CARE datasets (2D output and 3D output).

Fig. S16. Four-dimensional cardiac image reconstructions on the timeline.

Fig. S17. Line-scanning light sheet fluorescence imaging system schematic diagram.

Fig. S18. Scattering suppression results of LS-LSFM.

Fig. S19. Characterization results of the developed imaging platform based on LS-LSFM.

Fig. S20. *In vivo* cardiac dynamics restoration results at 60 h.p.f.

Fig. S21. Restoration results using CNNs networks.

S22. Experiment settings of *in vivo* zebrafish heartbeat imaging and training datasets.

**Other Supplementary Materials for this manuscript include the following:**

Movie S1. Irregular cardiac occurs with an instantaneously increased laser power (i.e. high to 0.3 mW) after two normal cycles with low laser power of 0.1 mW.

Movie S2. Whole cardiac dynamics movie before and after UI-Trans restoration (48 h.p.f.).

Movie S3. Whole cardiac dynamics movie before and after UI-Trans restoration (30 h.p.f.).

Movie S4. Whole cardiac dynamics movie before and after UI-Trans restoration (120 h.p.f.).

**Table S1.** **CNNs with transformer on low-level CV tasks.**

There is an increasing interest in deep learning research to explore various combinations of Transformer and CNN architectures for diverse image processing applications. Especially, there are a number of new works in recent months (some currently under review), each offering different advantages on its designed purposes, as listed in Table S1. Yet, we believe that further exploration is desirable in the development of more balanced hybrid architectural design and more refined training process to address the challenges posed by complex noise-scattering-coupled degradation.

Table S1. CNNs with transformer on low-level CV tasks.

| *Model Name* | *Base* | *Combination*  *Ways* | *Tasks* | *Application Areas* |
| --- | --- | --- | --- | --- |
| UI-Trans (Ours) | (double Encoder – decoder)  U-net | Transformer encoder branch | Restoration  (noise-scattering-coupled degradation) | Light sheet microscopy |
| CNNT[1] | (Encoder-decoder)  U-net | CNN-based Transformer block | denoising | Wide-field, two-photon and confocal microscopy |
| CTNet[2] | - | Multi block crossing | denoising | mobile digital devices |
| GVTNet[3] | (Encoder-decoder)  U-net | GVTOs block | Label-free prediction/denoising/ | microscopy (CARE datasets) |

**Fig. S1. SNR characterization of the developed imaging system.**

Theoretically, under conditions where shot noise predominates and photon generation is proportional to exposure, the SNR improves in proportion to the square root of the exposure. In this case, when calculating the SNR in dB scale, 3000 times the light exposure could lead to the SNR improvement of 17.3 dB, as following equations.

$SNR=\frac{\lambda}{\sqrt{\lambda}}$, where *λ* represents the number of photons received

$SNR(dB)=10\times{log}_{10}(\frac{\lambda}{\sqrt{\lambda}})$)

However, it should be noted that this relationship only holds true when the increase in exposure leads to a linear rise in the number of photons captured, thereby enhancing the SNR as the dominant noise source—shot noise—increases only with the square root of the photon count. Unfortunately, none of the conditions were satisfied in this study and therefore, the SNR only improves ~8.9 dB due to the ~3.0 dB loss for the former one and ~5.7 dB loss for the latter one.


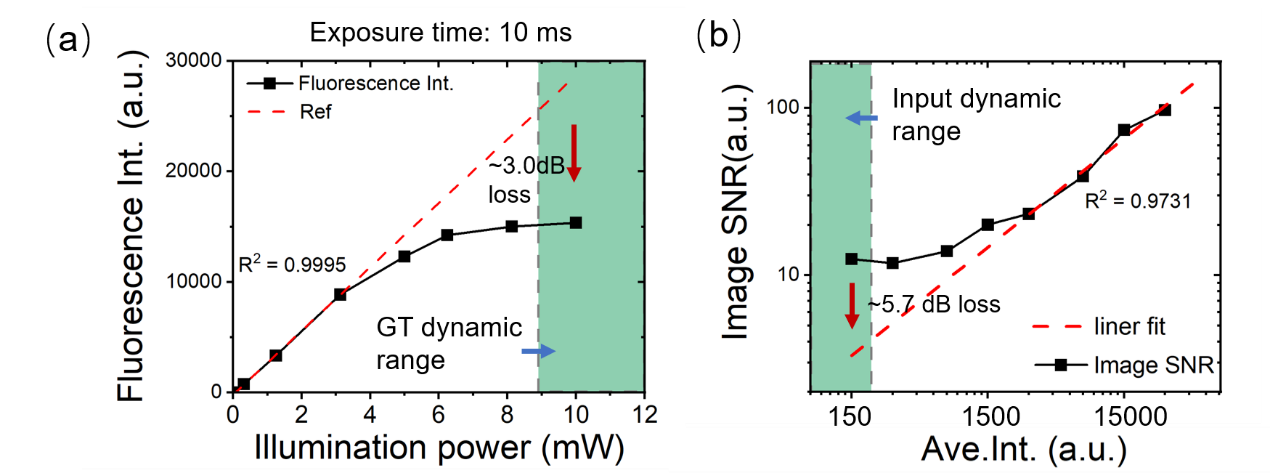


Fig. S1. SNR characterization of the developed imaging system. (a) The relationship between the fluorescence intensity and the illumination power on zebrafish (TG: kdrl-EGFP). (b) The relationship of the acquired image SNR and the received signal intensity (by using different exposure time).

For the former condition, since fluorescence is used as the imaging contrast and at high illumination powers, the complex fluorescence process could be affected by photobleaching or non-linear effects (e.g. saturation, thermal effect), The number of fluorescence photons generated from complex biological organisms does not always increase linearly with the intensity of excitation, especially in our case with a 100 times difference in illumination power (i.e. Low: 0.1 mW; High: 10 mW). To experimentally show the relationship between the generated fluorescence photons and the illumination power in our study, the light sheet slice images of zebrafish (TG: kdrl-EGFP) were collected under different illumination powers (i.e. from 0.1 mW to 10 mW) with the same exposure time (i.e. 10 ms) at the same slice position of the sample. As shown in the Fig.S1 (a), at illumination powers below approximately 3 mW, the fluorescence signal intensity maintains a linear relationship with the illumination power. However, as illumination power exceeds this threshold, the emission efficiency of the fluorophore begins to saturate, leading to a gradual reduction in the rate of increase in emission intensity. By the time the illumination power reaches 10 mW (i.e. the power used for the GT data acquisition in this study), the emission intensity of the fluorophore approaches its maximum. Due to the loss of this linear relationship, the increase in illumination power used in this study results in an approximate 3 dB reduction in SNR improvement.

For the latter condition, considering the noise in our imaging system is rather complex (i.e. deviated from the predomination of shot noise), especially in low-light detection [4, 5] using a sCMOS camera with a built-in function of 'Real Time Application Optimization' which automatically enhances the electronical signals at low signal levels, in order to illustrate the relationship between the SNR and the detected signal levels in our study, we characterized the SNR-exposure curve of the camera (Prime BSI) by uniformly illuminating with white light on the camera fixed on the Olympus IX70 microscope using exposure times of 1 ms, 2 ms, 5 ms, 10 ms, 20 ms, 50 ms, 100 ms and 200 ms. Fig.S1 (b) shows the SNR variations of the camera at different detected signal levels (indicated by black points), while the red dashed line represents the slope that a shot noise dominated system should follow at this logarithmic coordinate. It is noted that in our imaging system, the SNR does not consistently adhere to square root linearity with respect to detected signal levels. This deviation typically occurs when the received photon signal is excessively weak. When the detected signal is less than 1600 a.u. (using 16 bit), the decrease of SNR becomes negligible. This can be partially explained by the fact that the Prime BSI camera automatically performs the 'Real Time Application Optimization' function at low signal. In our study, the low-SNR acquisition falls within this range of low signal levels, resulting in an approximate 5.7 dB reduction in SNR improvement.

**Fig. S2. Restored results using CARE, RCAN and UI-Trans network via maximum intensity projection (MIP) method.**

Maximum intensity projection (MIP) is a rendering technique by creating a 2D image from a 3D dataset through projecting the maximum value of each voxel along a specified direction, which means it visualizes only the brightest structures in the view path, ignoring less intense structures. This projection method is commonly used in high-contrast volumetric data visualization due to its vivid display, but it is prone to some visual misunderstandings for biological samples especially with multi-layer structures. Alternatively, surface blend is also a commonly used technique in 3D visualization of volumetric data by creating a 3D representation of surfaces within the volume based on a threshold intensity. It can provide a more realistic 3D view of the surfaces in the data and display complex structures’ spatial relationships more intuitively. We note that while both MIP and surface blend have been widely used for 3D visualization, they have its own specific uses, advantages, and limitations. More importantly, these approaches are rendering methods for 3D visualizations of volumetric data, yet cannot represent the comprehensive analysis of the volumetric data. In this study, we have provided 3D visualizations using both MIP and surface blend methods.


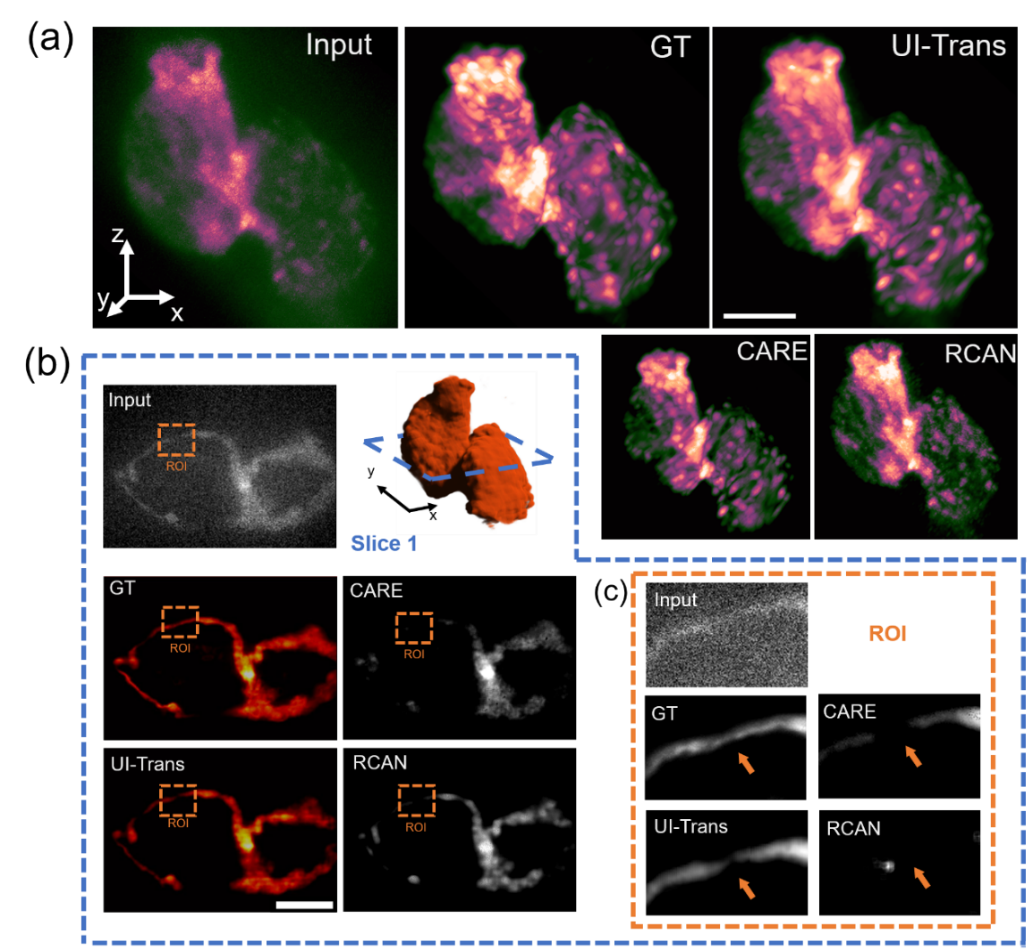


Fig. S2. Restored results using CARE, RCAN and UI-Trans network. (a) 3D visualizations via maximum intensity projection (MIP) of a whole zebrafish heart acquired with low-quality LSFM (input), high-quality confocal LS-LSFM (GT) and the restored results using CARE, RCAN and UI-Trans network. (b) 2D slice images in x-y plane. (c) 2D zoomed-in images in the ROI (red rectangle) of (b).

While Figure 2(a) in the manuscript shows the 3D visualization via surface blend method, the comparison images via MIP rendering were also plotted (as shown in Fig. S2(a)). The image restoration results of UI-Trans, RCAN, and CARE can also be evaluated from 2D slice images. Fig. S2 (b) and show slices of the 3D heart data depicted in Fig. S2 (a), demonstrating an effective restoration of the slender myocardial layer (as shown in Fig. S2 (b) and (c)) which preserves intricate details that are lost in the output image of RCAN and CARE.

**Fig. S3 and S4. Quantitative analysis based on evaluation metrics on each individual data in the validation set.**

To illustrate the overall performance of UI-Trans compared with CARE and RCAN on individual data in the validation set, the differences in normalized root mean square error (NRMSE), Pearson correlation, peak signal-to-noise ratio (PSNR) and structural similarity index (SSIM) between the UI-Trans-restored images and the CARE- and RCAN-restored images in all 40 3D images in the validation set were calculated (as shown in Fig. S3 and S4), straightforwardly exhibiting the improvement of UI-Trans in each data using these evaluation metrics. It is also noted that the advantage of UI-Trans is prominent in cases where the degradation is more complex. This can be explained by the combination of ViTs and U-Net providing a comprehensive view of both local and global image features as well as an enhanced feature extraction capability.


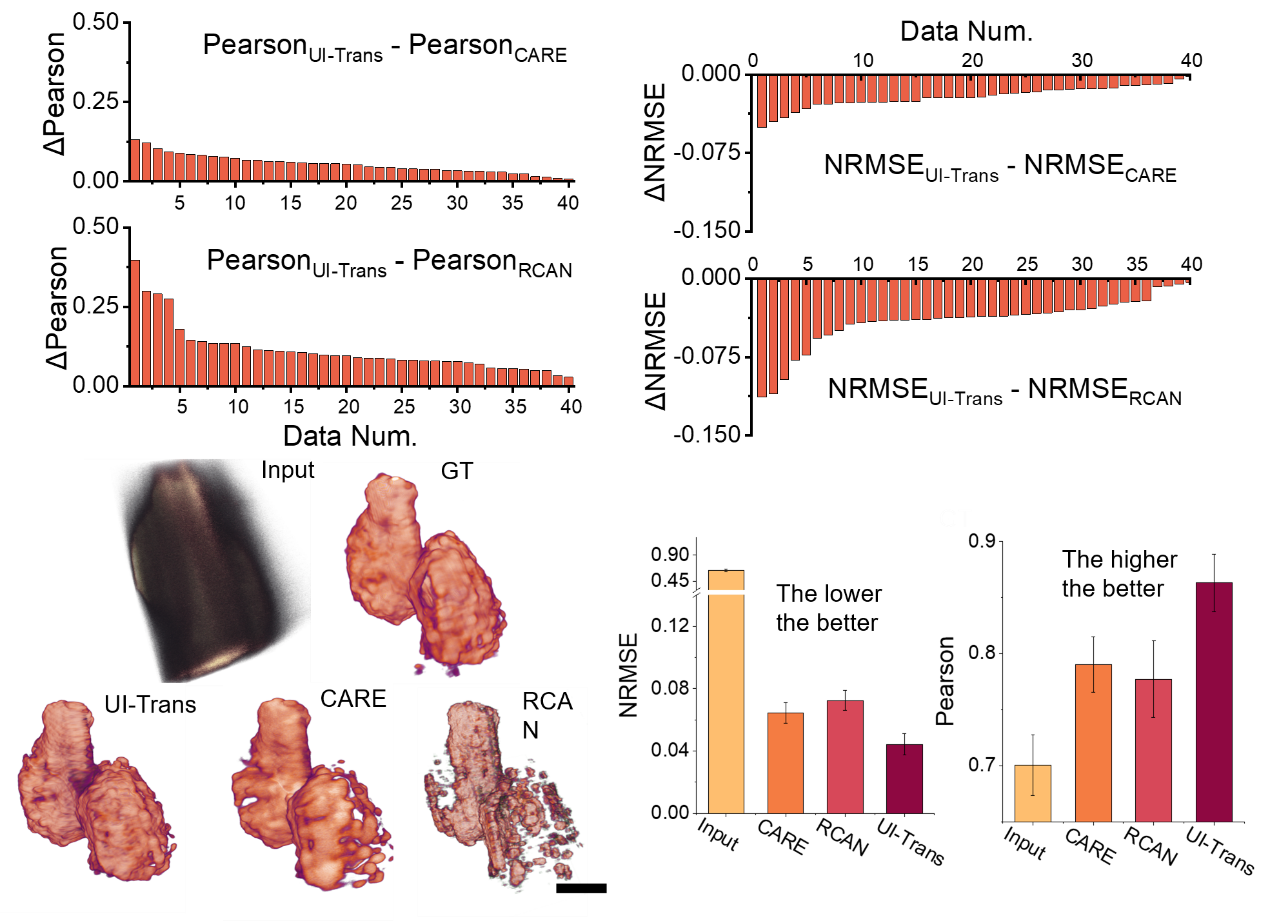


Fig. S3. The normalized root means square error (NRMSE) and Pearson coefficient differences between UI-Trans, CARE and RCAN networks. (a) The Pearson coefficient and NRMSE differences on all 40 3D validation data. (b) Replotted 3D images in Fig. 2(a) and the corresponding calculated Pearson coefficient and NRMSE.


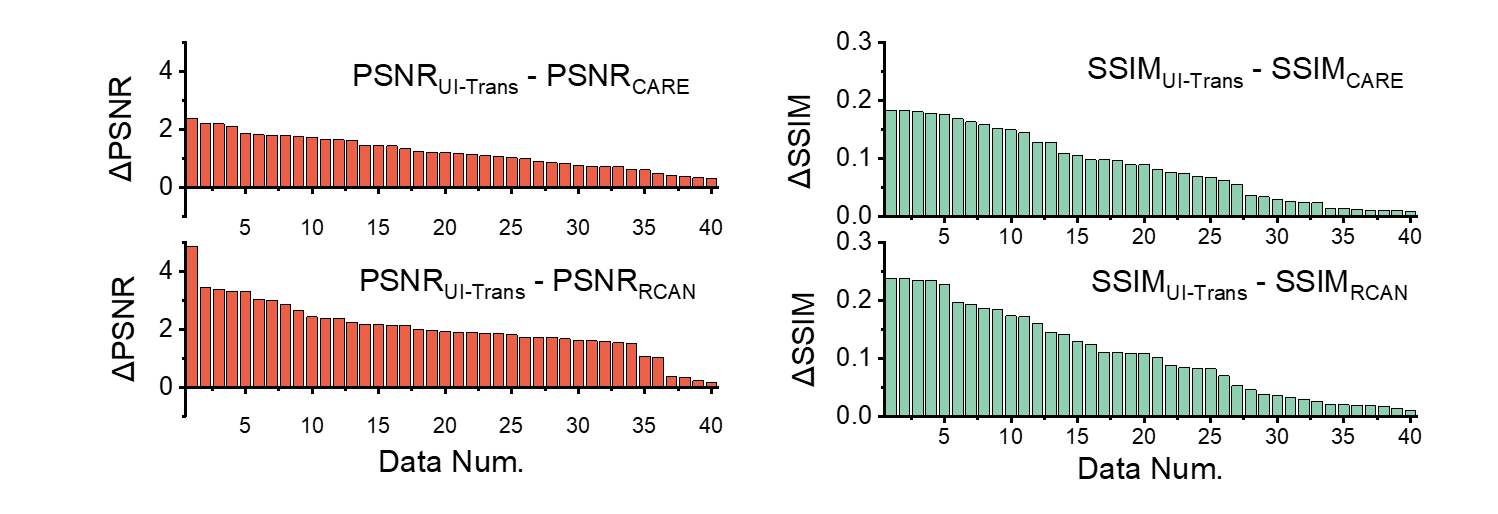


Fig. S4. The calculated peak signal-to-noise ratio (PSNR) and structural similarity index (SSIM) differences between UI-Trans, CARE and RCAN networks on all 40 3D validation data.

**Fig. S5. Scattering-alleviating investigation using 2D image comparisons.**


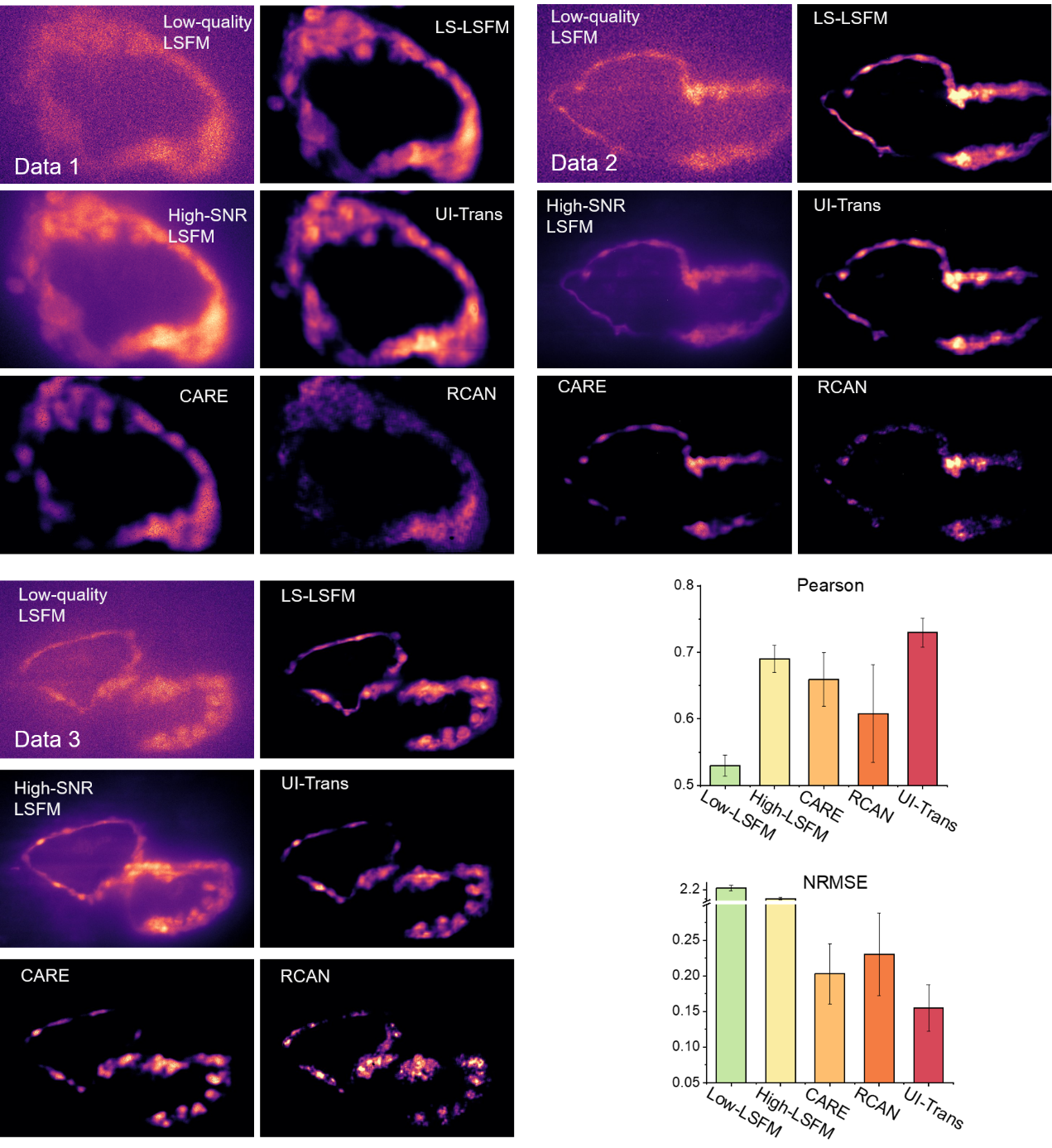


Fig. S5. Comparison of 2D slice images acquired from a low-quality conventional LSFM (Input), high-SNR conventional LSFM, confocal LS-LSFM (GT), and the restored results from UI-Trans, CARE and RCAN respectively, as well as the corresponding Pearson coefficient and NRMSE.

We provide more comparison results of 2D slice images acquired from a low-quality conventional LSFM (Input), high-SNR conventional LSFM, confocal LS-LSFM (GT), and the restored results from UI-Trans, CARE and RCAN respectively (as shown in Fig. S5). In addition to the metrics (i.e. contrast enhancement and SNR in Fig. 3 in the manuscript) used for the evaluation of the restoration quality confronting complex scattering-noise-degradation, the Pearson coefficient and NRMSE were also calculated.

**Fig. S6. 2D slice images of Input and UI-Trans-restored data of *in vivo* heartbeat imaging.**


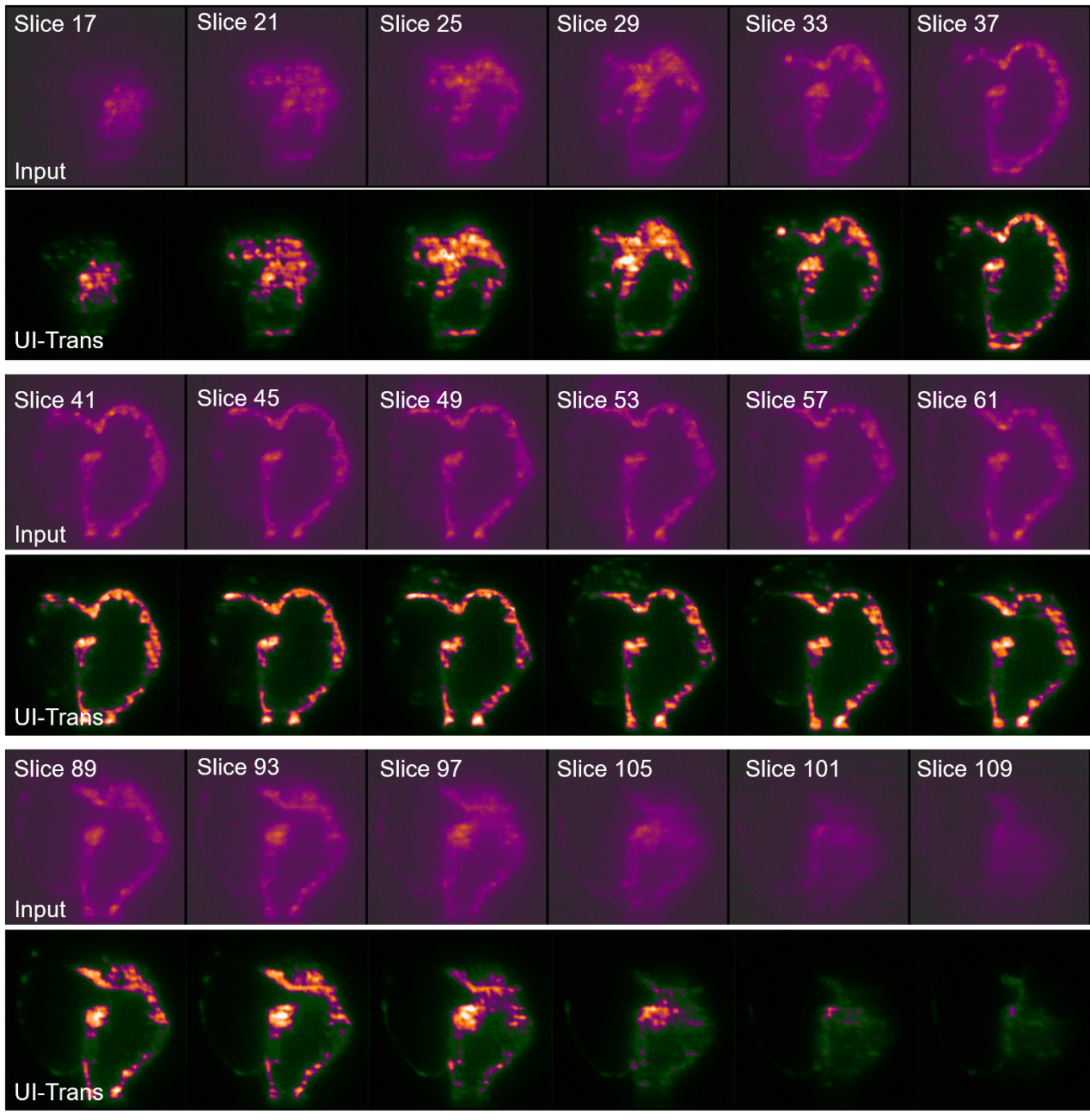


Fig. S6. 2D slice images of Input and UI-Trans-restored data of *in vivo* heartbeat imaging at different spatial locations.

Fig. S6 shows a series of 2D slice images of Input and UI-Trans-restored data of *in vivo* heartbeat imaging, illustrating that the restored image preserves structural and detail fidelity.

**Fig. S7. 2D slice images of Input and restored data using pre-trained UI-Trans, CARE and RCAN for *in vivo* heartbeat imaging.**


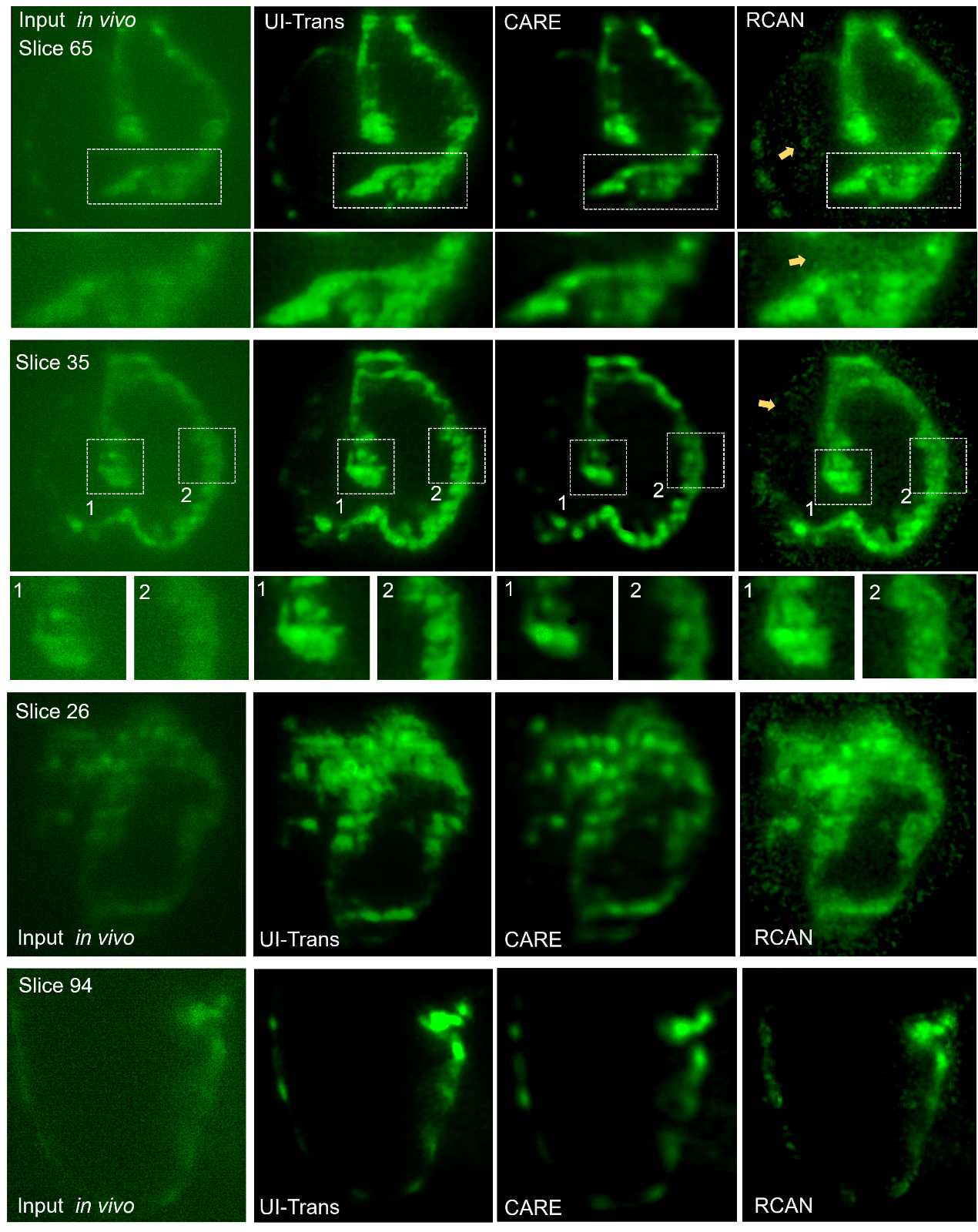


Fig. S7. 2D slice images of Input and restored data using pre-trained UI-Trans, CARE and RCAN for *in vivo* heartbeat imaging.

Fig. S7 shows more 2D slice images at different spatial locations from pre-trained networks using CARE, RCAN and UI-Trans, demonstrating the higher performance of UI-Trans in terms of denoising, scattering alleviating and detail preservation. These detailed 2D slice images agree well with the evaluation metrics. It is noted that in the restored results, RCAN performs well on strong signals, but it cannot distinguish between noise and weak signals, resulting in noticeable blurring its restored image. This could adversely affect the SNR and contrast enhancement. CARE exhibits denoising capabilities on *in vivo* data, but its effectiveness in restoring image details is constrained, resulting in the decrease of visual perception.

**Fig. S8. The Input, GT and UI-Trans-restored images at relatively higher SNR level in *ex vivo* datasets.**


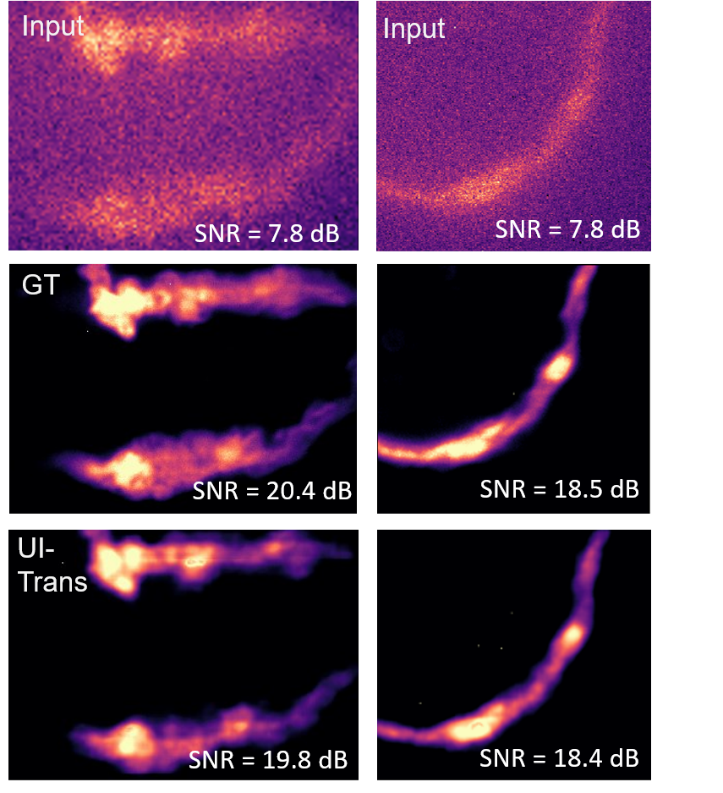


Fig. S8. The Input, GT and UI-Trans-restored images at relatively higher SNR level in *ex vivo* datasets (Input ~7.8 dB; GT ~19.4 dB and UI-Trans-restored images ~ 19.1 dB).

The SNR of 3D images can be greatly affected by different samples acquired at different angles. Therefore, the SNR of the images is largely varied in the training dataset (low-quality conventional LSFM ranging from 4.5 -8.1 dB and high-quality LS-LSFM ranging from 12.5 dB-20.4 dB). Fig. S8 shows The Input, GT and UI-Trans-restored images at relatively higher SNR level in *ex vivo* datasets, demonstrating that the GT of certain samples did achieve SNR higher than 17 dB as well as the corresponding restored results.

**Fig. S9. The input and output images of the networks in the ablation experiment.**


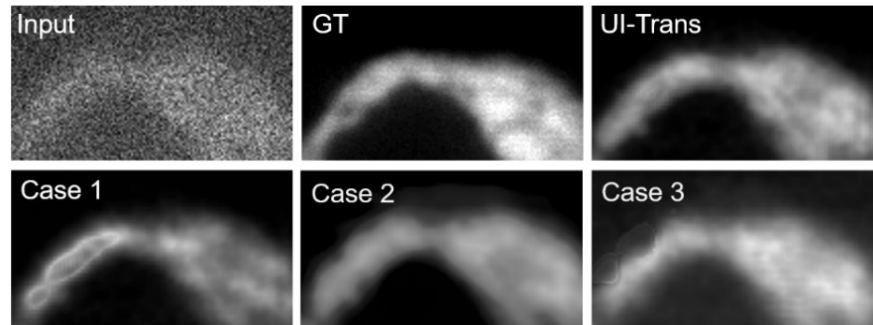


Fig. S9. The input and output images of the networks in the ablation experiment. UI-Trans: final framework used in our work; Case 1: based CNN only; Case 2: CNN incorporating Transformer blocks in both encoder and decoder branches; Case 3: UI-Trans with MAE loss only.

**Table. S2. The ablation experiment results.**

Table. S2. The ablation experiment results. UI-Trans: final framework used in our work; Case 1: based CNN only; Case 2: CNN incorporating Transformer blocks in both encoder and decoder branches; Case 3: UI-Trans with MAE loss only.

|  | U-shape CNNs | Transformer encoder | Transformer decoder | Loss | SSIM | PSNR (dB) |
| --- | --- | --- | --- | --- | --- | --- |
| UI-Trans  (benchmark) | **√** | **√** | - | MAE +perceptual | **0.68** | **26.47** |
| Case1 | √ | - | - | MAE+ perceptual | 0.63 | 25.57 |
| Case2 | √ | **√** | √ | MAE +perceptual | 0.58 | 25.46 |
| Case3 | **√** | **√** | - | MAE | 0.68 | 24.80 |

The functionality of the key components of the developed UI-Trans network was analyzed, and the efficacy of multi-loss joint optimization strategy was evaluated through the ablation experiments (results shown in Fig.S9 and Table.S2) by focusing on the Transformer block and its position as well as the loss function, as well as the trade-off between performance and computational burden using SSIM, PSNR and the total number of network parameters. Our UI-Trans network was set as the benchmark and case 1-3 represented the basic architecture without Transformer blocks using joint-loss, the architecture with Transformer blocks in both encoder and decoder branches using joint-loss and UI-Trans network only using pixel-level MAE loss respectively. It is noted that there is a notable reduction in performance (-5% SSIM and -0.90 dB) and restored images by using the basic U-type CNN architecture. Moreover, the image analysis reveals that this basic U-net tends to overly emphasize local details and to generate details with the deviation of authenticity. This issue is addressed by UI-Trans via introducing Transformer blocks in the encoder branch, showing improved comprehending complex global features while extracting sufficient details in feature maps. However, incorporating Transformer blocks in both encoder and decoder branches results in a drastic loss of detailed information (-10% SSIM and -1.01 dB), underscoring the Transformer module's emphasis on the overall feature distribution within the network. Furthermore, it is noted that UI-Trans network with only MAE loss for training would degrade the performance by a large margin (-1.67 dB) as well as the visualization, demonstrating the higher efficiency of multi-loss joint optimization against the commonly-used MAE loss for microscopy images.

**Fig. S10. Performance evaluation of UI-Trans using a reduced image set for training.**


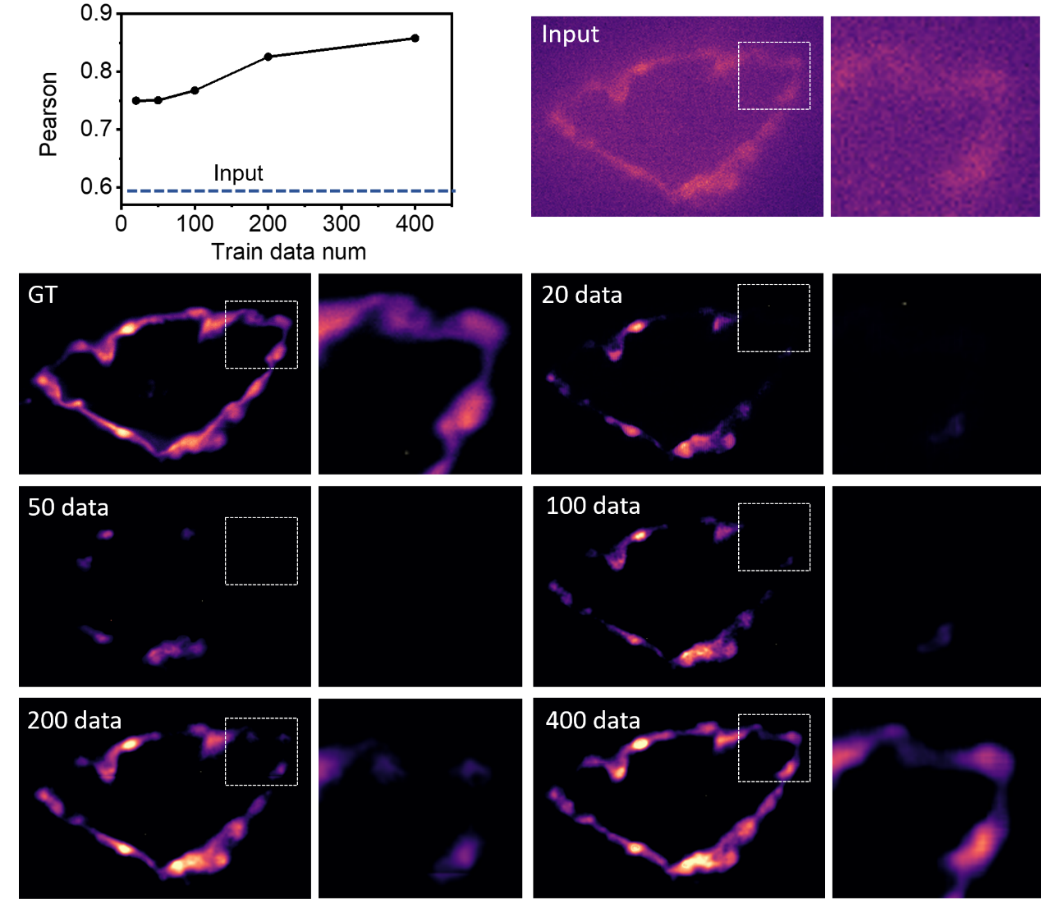


Fig. S10. Performance evaluation of UI-Trans using a reduced image set for training including 20, 50, 100 and 200 data compared with 400 data.

A number of experiments were conducted to analyze the effects of the amount of training data for UI-Trans performance in terms of denoising and detail preservation, and the results are shown in Fig. S10. It is noted that a significant enhancement in image quality can be achieved with a modest dataset of 20 and 50 samples. However, further increases in the training data can lead to even higher performance, especially in terms of detail preservation.

**Fig. S11. Restoration results using classical algorithms.**


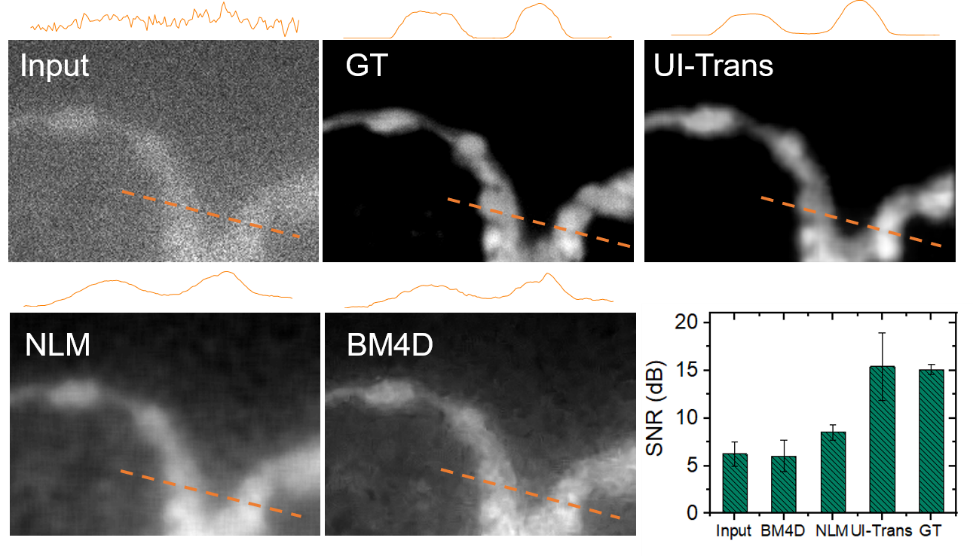


Fig. S11. Restoration results using classical algorithms. (a) Low-quality LSFM image restoration with classical denoising algorithms (non-local means (NLM) and BM4D) and deep-learning method (i.e. UI-Trans), along with the cross-section intensity profile of the orange line. (b) SNR of NLM, BM4D, input and UI-Trans.

Classical algorithms (including BM4D and non-local means (NLM)) were used for the enhancement of low-quality LSFM images and the results were compared with the deep learning methods. It is noted that the developed UI-Trans significantly outperformed classical algorithms in both visualization and quantitative metrics on the datasets in this study. Nevertheless, classical algorithms retain a number of advantages, including consistency between input and output and adaptability across diverse categories of images. The success of deep learning models critically depends on a sufficient amount of high-quality training data.

**Fig. S12. The trade-offs of LSFM imaging between imaging speed, light exposure and image quality.**

*
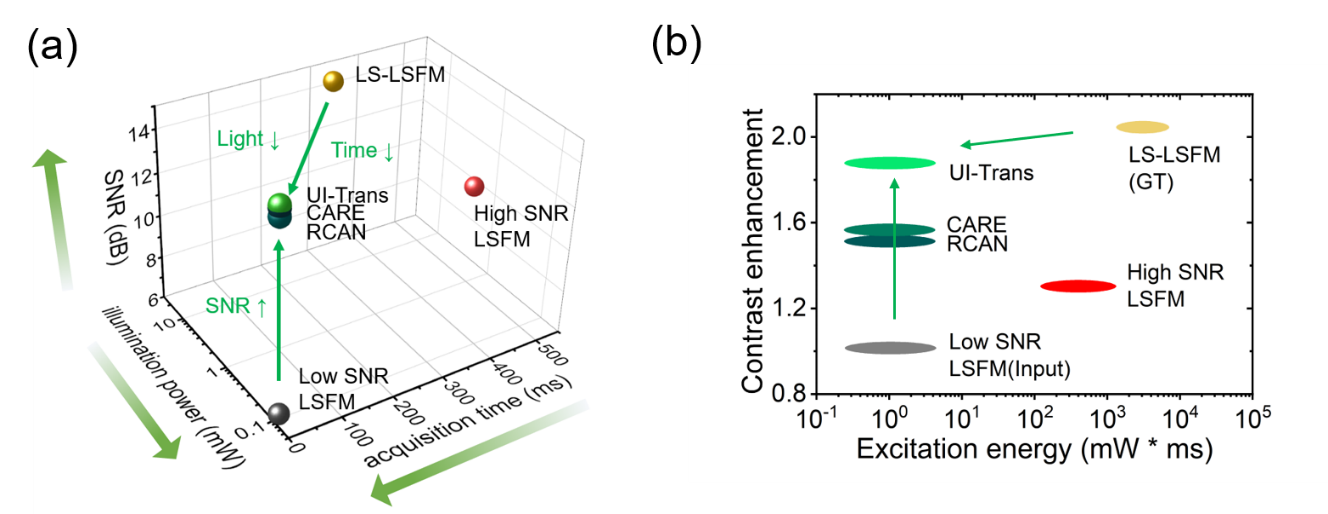
*Fig. S12. The trade-offs of LSFM imaging between imaging speed, light exposure and image quality. (a) The trade-offs between acquisition time, illumination power and image SNR of low-quality LSFM, high-SNR LSFM, confocal LS-LSFM and deep-learning-enhanced LSFM; (b) The trade-off between light exposure on the same and contrast enhancement of different imaging strategies.

The experimental settings in terms of imaging performance were evaluated for different methods (i.e. low-quality conventional LSFM, high-SNR conventional LSFM, confocal LS-LSFM and three deep-learning-enhanced LSFM modalities) in terms of illumination power, acquisition time, total light exposure, SNR and contrast enhancement. In previous studies such as CARE and RCAN for the denoising task, the training data is usually acquired using several laser intensities/exposure times, and the high-SNR images are served as ground truth. However, *in vivo* imaging of mesoscopic organisms often suffers from varying noise, uneven scattering, and complex moving biological structures, all of which result in highly complex degradations in image quality. As shown in Fig. S12, confocal LS-LSFM could effectively mitigate scattering that cannot be eliminated by simply increasing the illumination and exposure of conventional LSFM (i.e. high-SNR conventional LSFM), presenting significantly improved contrast and SNR. More importantly, UI-Trans-enhanced LSFM achieves competitive high-quality images with confocal LS-LSFM but requiring less than 0.03% of the light exposure and 3.3% of the acquisition time. This reduction in light exposure and acquisition time is particularly crucial for long-term live imaging of fast processes, since it not only effectively minimizes the adverse effects of prolonged light exposure on the subject organisms but also facilitates rapid imaging with low-intensity light.

**Fig. S13. Exploration on further analysis for heartbeat rhythm information.**


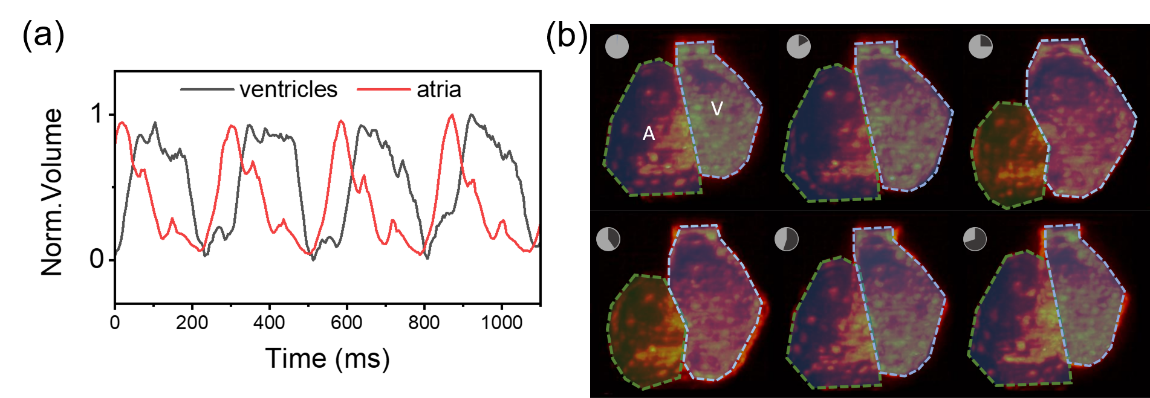


Fig. S13. Exploration on further analysis for heartbeat rhythm information. (a) Normalized volumetric curve of the zebrafish ventricle and atrium over time. (b) Schematic diagram of changes in the heart/atrium during one heartbeat cycle. A, atrium; V, ventricle.

To further explore the potential image processing on the enhanced images of zebrafish cardiac images, a threshold-based morphological operation method was employed to quantify the volumetric alterations in the atrial and ventricular chambers throughout ~4 cardiac cycles with the restored images of zebrafish heartbeats, and the results are shown in Fig. S13. The method is performed as following steps. Initially, a manual segmentation on the UI-Trans-restored images was conducted to isolate dynamic images of the atrium and ventricle separately. Subsequently, adaptive threshold segmentation was applied to each slice of the atrium/ventricle images to generate binary images. Then, the binary images were processed sequentially using erosion, dilation, skeleton extraction, and closed area filling to calculate the cavity area in each slice. These areas across all slices were summed to determine the atrium/ventricle volume, which is subsequently normalized. Finally, performing statistical analysis on the volume data from all time points enables the construction of the heart rhythm curve. The results demonstrate that the heart volume exhibits a consistent and reliable temporal variation, suggesting the potential of UI-Trans-based image restoration for further investigations into cardiac dynamics.

**Fig. S14 and S15. Applicability exploration of UI-Trans on CARE datasets (2D output and 3D output).**

To explore the applicability of UI-Trans for other organisms on other microscopy techniques, a number of online accessible imaging data on *Tribolium castaneum* embryos, *S. mediterranea* flatworm and *D. melanogaster* drosophila wing (i.e. from CARE online content, acquired with confocal scanning microscopy and spinning disk microscopy) were used as additional multiple examples, and the restored results (as shown in Fig. S14 and S15) demonstrate that the developed UI-Trans can perform well on a wide range of application with proper adaptation, especially for more complex and composite tasks (e.g. the complex image degradation problem of miscellaneous varying noise and uneven scattering in this study). For instance, the flatworm (*S. mediterranea*) is a model organism for studying tissue regeneration, which is extremely sensitive to even moderate amounts of laser light, easily exhibiting muscle flinching at relatively higher illumination levels even when anesthetized. Consequently, the live imaging of *S. mediterranea* is challenging to be intractable. With the enhancement of UI-Trans, it is able to turn the low-SNR unusable data of live-cell imaging of *S. mediterranea* to high-quality time-lapse data for biological investigations. We believe future explorations of joint-training with different microscopy techniques on various organisms will further broaden the applicability of UI-Trans to address more complex biological imaging tasks.


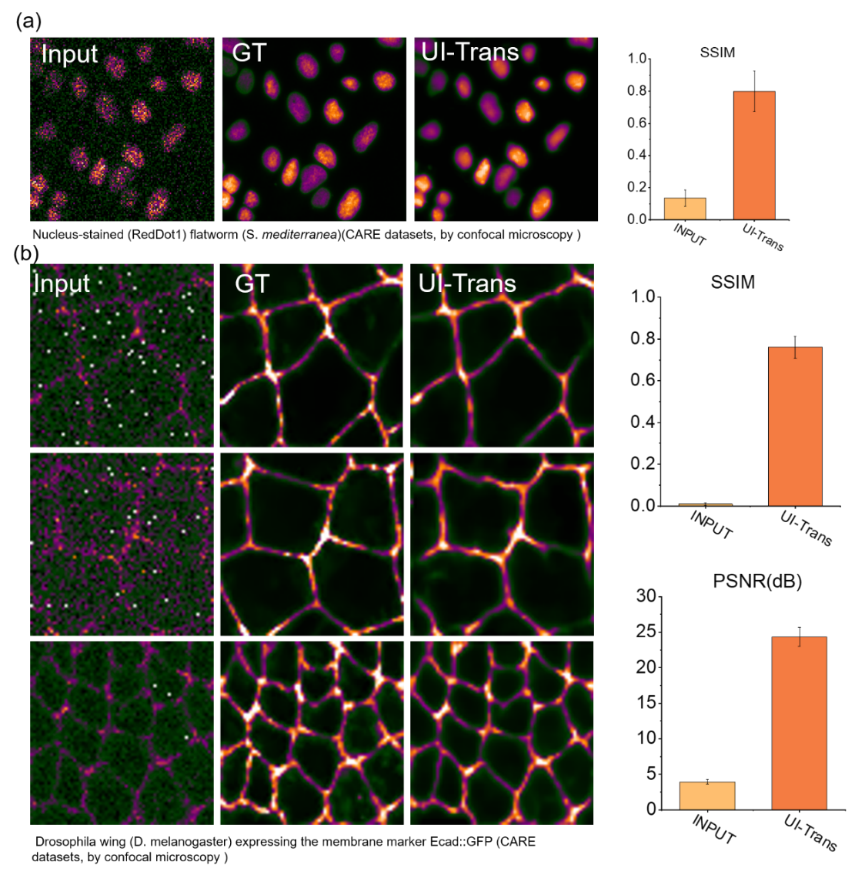


Fig. S14. Applicability exploration of UI-Trans on CARE datasets with 2D output. (a) Input data (2D) and restorations (2D) for nucleus-stained (RedDot1) flatworm (*S. mediterranea*) (acquired by confocal scanning microscopy). (b) Input data (3D) and restorations (2D projection) on E-cadherin-labeled fly wing data (acquired by spinning disk microscope).


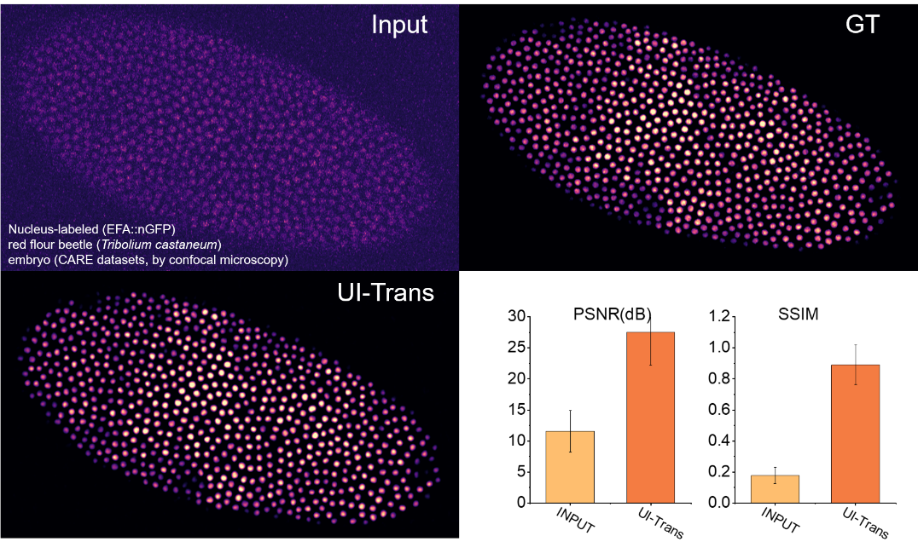


Fig. S15. Applicability exploration of UI-Trans on CARE datasets with 3D output. Input data (3D) and restorations (3D) for a nucleus-labeled (EFA:nGFP) red flour beetle (*Tribolium castaneum*) embryo (acquired by confocal scanning microscopy).

**Fig. S16. Four-dimensional cardiac image reconstructions on the timeline.**


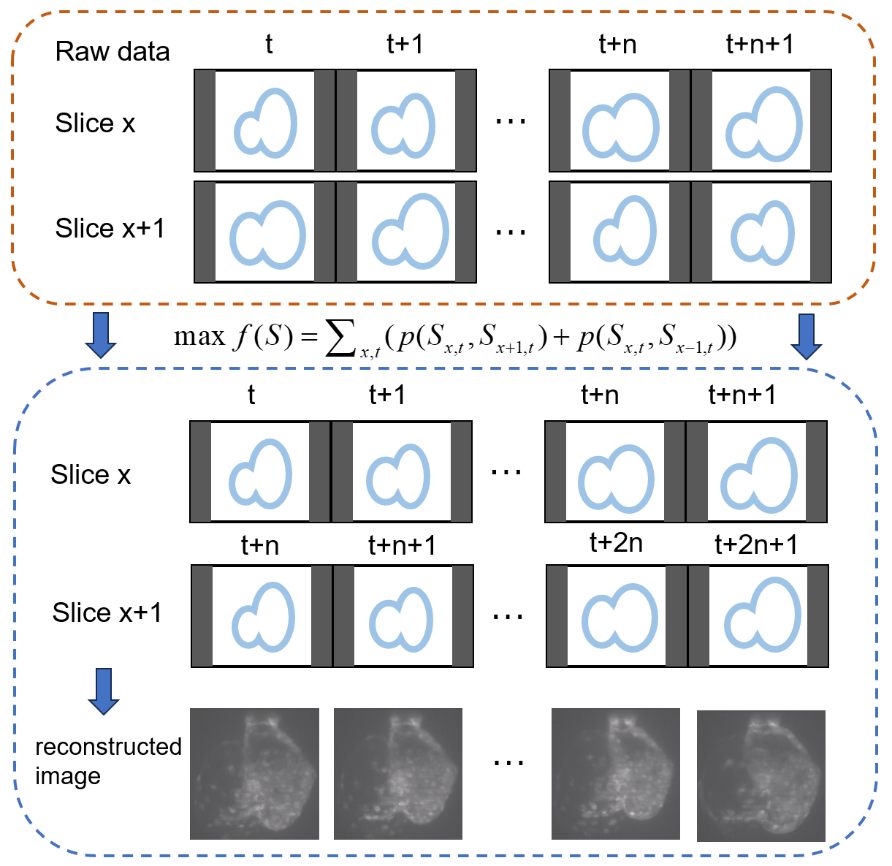


Fig. S16. Four-dimensional cardiac images reconstruction on the timeline. Adjusting the time phase of all slices with particle swarm optimization algorithm to maximizes the optimization function, which is the sum of the Pearson correlation coefficient between all adjacent slices.

Direct 4D LSFM imaging is challenging for zebrafish heartbeats due to its extremely short heartbeat cycle, lasting for only hundreds of milliseconds. Instead, the three-dimensional cardiac dynamics can be reasonably reconstructed by synchronizing the movie stacks post-acquisition (as shown in Fig. S16). This method includes two parts: the time-phase-images acquisition of each slice and reconstruction. Firstly, the time-phase-images acquisition protocol includes acquiring 1000 frames at each slice position and the total number of slices is determined by the cardiac thickness. In this demonstration, the exposure time of each frame was set to a typical 3 ms with a laser intensity of 0.1 mW, achieving a rapid frame rate of 332 fps. *In vivo* 4D imaging of a heartbeat typically took approximately 10 to 20 minutes and the heartbeat of zebrafish remained relatively stable during this period, providing a reliable basis for reconstruction. Subsequently, 4D heartbeats movie could be obtained by aligning the phase of each slice in the movie stack and performing a 3D reconstruction on the timeline. The particle swarm optimization algorithm was employed to match and reconstruct the phase of the heartbeat, assuming that the Pearson correlation coefficient between adjacent slice images with the same phase is maximized. The algorithm began with an initial phase value for a slice and employed a population optimization algorithm to determine the optimal solution for the phases of other slices in close proximity. It iteratively calculated the overall optimal solution for the phases until it converged to the global optimal solution. The objective function was formulated as the sum of the Pearson correlation coefficients between each slice position and its adjacent and sub-adjacent slices, following

$$\max f(S)=\sum_{x,t} (p(S_{x,t},S_{x+1,t})+p(S_{x,t},S_{x-1,t}))$$

where *S* is the slice image, *x* and *t* represent the space and time phase of the slice image *S* respectively, and *p* represents calculating the Pearson correlation coefficient of two images. The reconstructed 3D heart images captured at different time points were divided into 256×256×256, and subsequently fed into pre-trained networks.

**Fig. S17.** **Line-scanning light sheet fluorescence imaging system schematic diagram.**


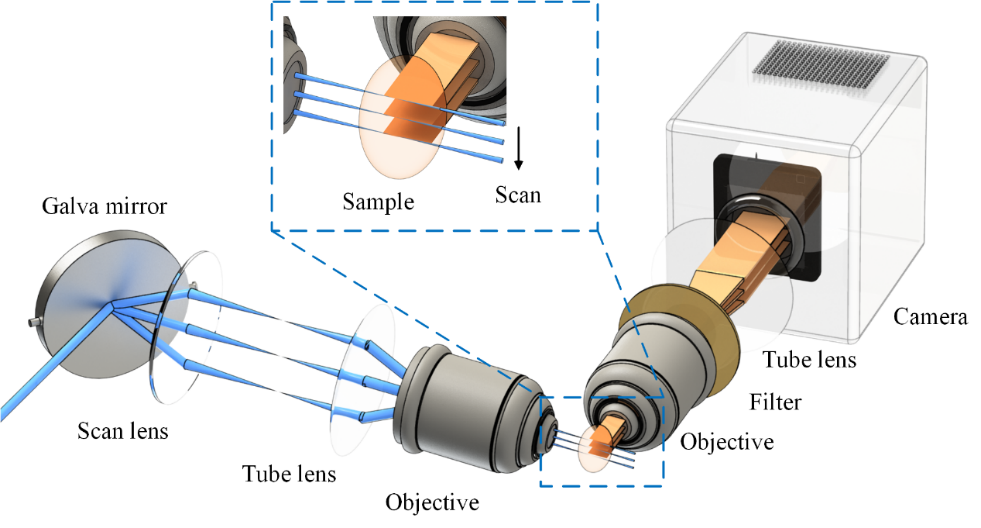


Fig. S17. Line-scanning light sheet fluorescence imaging system schematic diagram.

The LS-LSFM system employed the same optical setup as the LSFM, and this enables the rapid and straightforward generation of appropriate training data via the flexible switching between conventional LSFM and confocal LS-LSFM on the same imaging system. In the LSFM system, the galvo mirror oscillated at a high frequency to drive the beam and to form a light sheet. In the LS-LSFM mode of photons collection, however, the oscillation of the galvo mirror was slowed down to match the scanning imaging of the camera chip in rolling shutter mode. Consequently, the camera's line-by-line exposure was synchronized with the movement of the beam, ensuring that scattered light outside the beam was not captured by the camera. This synchronization in LS-LSFM imaging provided high-quality images with suppressed scattering.

**Fig. S18.** **Scattering suppression results of LS-LSFM.**

**
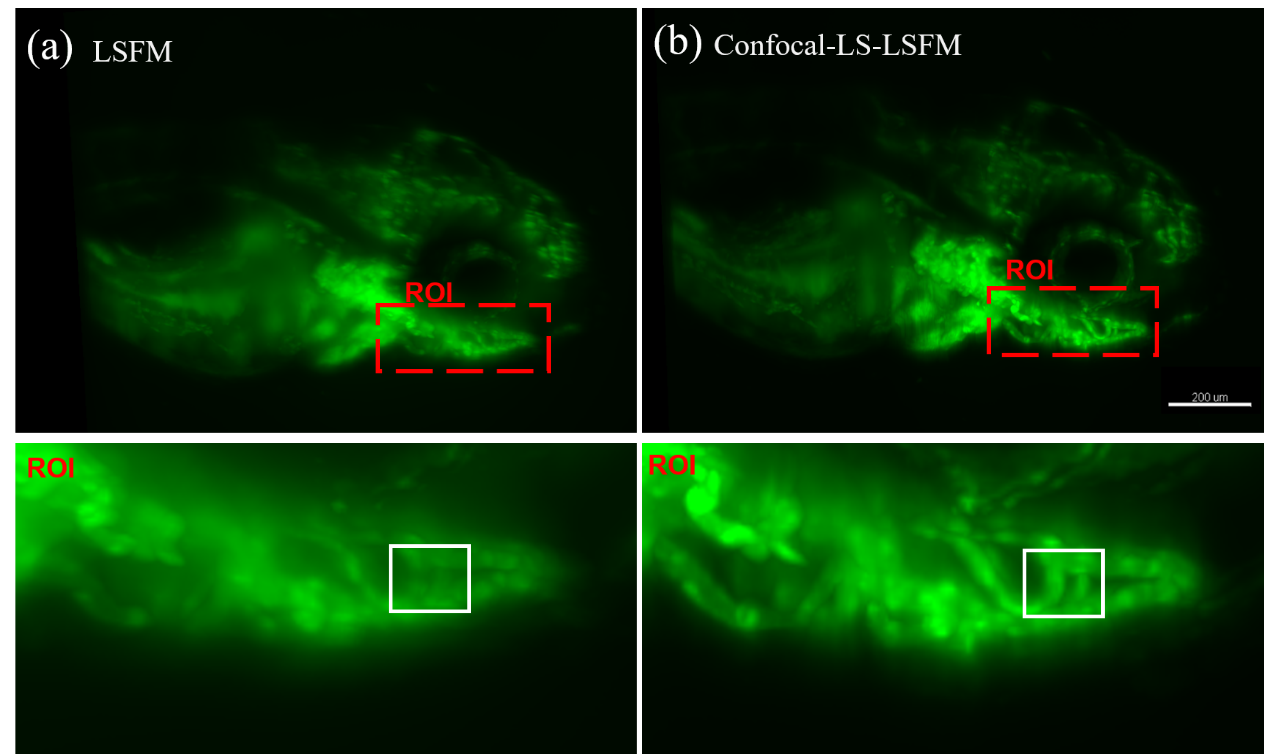
**

Fig. S18. 3D rendering images of a fixed zebrafish (5 d.p.f. Tg (kdrl: EGFP)) using (a) LSFM and (b) LS-LSFM respectively. Scale bar: 200 μm.

To evaluate the image quality improvement of LS-LSFM in terms of scattering suppression, we performed 3D imaging on fluorescent protein transgenic zebrafish (Tg (KDRL: EGFP), China Zebrafish Resource Center) using LSFM and LS-LSFM, respectively. After anesthesia with 100 mg·L^-1^ MS-222 (tricaine, Sigma A5040) solution for 20 minutes, the zebrafish were embedded in agarose and fixed in the sample chamber. Imaging was carried out sequentially at the same location using LSFM mode (1 mW excitation power, 200 ms exposure time) and LS-LSFM mode (0.5 mW excitation power, 1 ms exposure time per line). As shown in the Fig.S18, LS-LSFM significantly mitigates the image degradation caused by scattering, revealing image details that would otherwise be obscured by scattering. The magnified view from the ROI (red dashed rectangle) demonstrates that the image collected with LSFM exhibits higher background from scattering, making it difficult to distinguish blood vessel edges. However, the LS-LSFM 3D visualization shows higher contrast, allowing clear distinction of two blood vessels that are indiscernible in the LSFM image (as shown in white box). This scattering suppression improves the image quality, and thus facilitating higher-quality training datasets.

**Fig. S19. Characterization results of the developed imaging platform based on LS-LSFM**


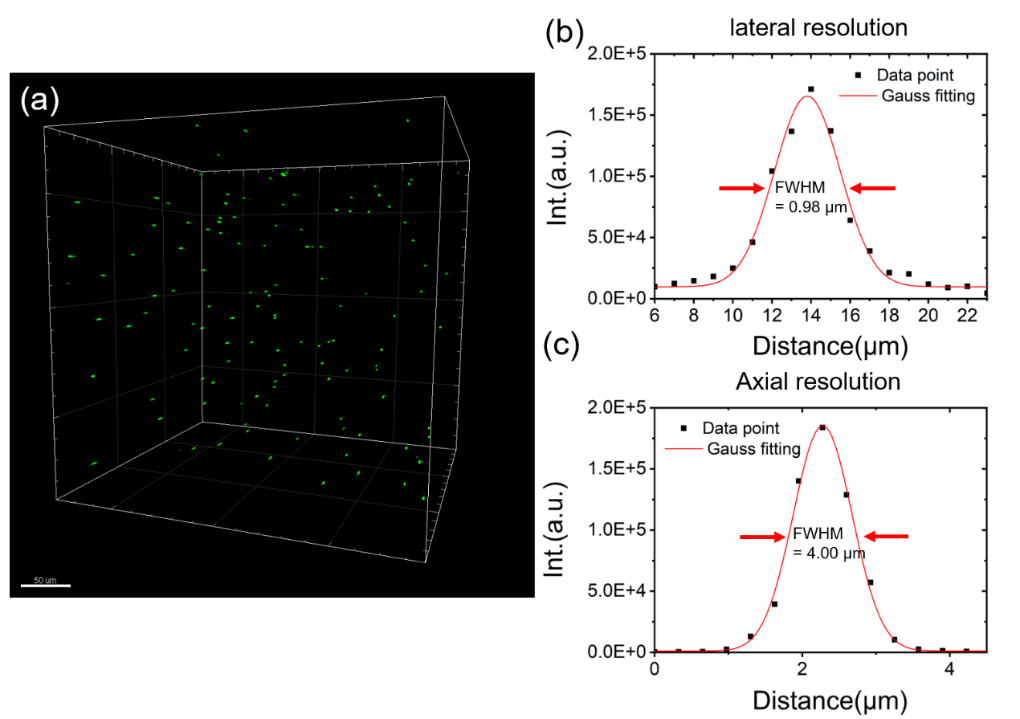


Fig. S19. Spatial resolution characterization results of LS-LSFM using the fluorescent bead phantom. (a) 3D images of fluorescent microbeads and intensity distribution along the (b) lateral and (c) axial direction of beads with Gauss fitting. Scale bar: 50 μm.

The fluorescent bead phantom consisting of a low concentration suspension of fluorescent beads (0.2 μm diameter, excitation/emission maxima at 505/515 nm) embraced in 1.5% agarose (A7002, SLBK5425V, Sigma Aldrich, St. Louis, MO, USA) were used to characterize the spatial resolution of LS-LSFM, exhibiting a lateral resolution of 0.98 ± 0.21 μm and an axial resolution of 4.00 ± 0.17 μm (as shown in Fig S19).

**Fig. S20. *In vivo* cardiac dynamics restoration results at 60 h.p.f.**


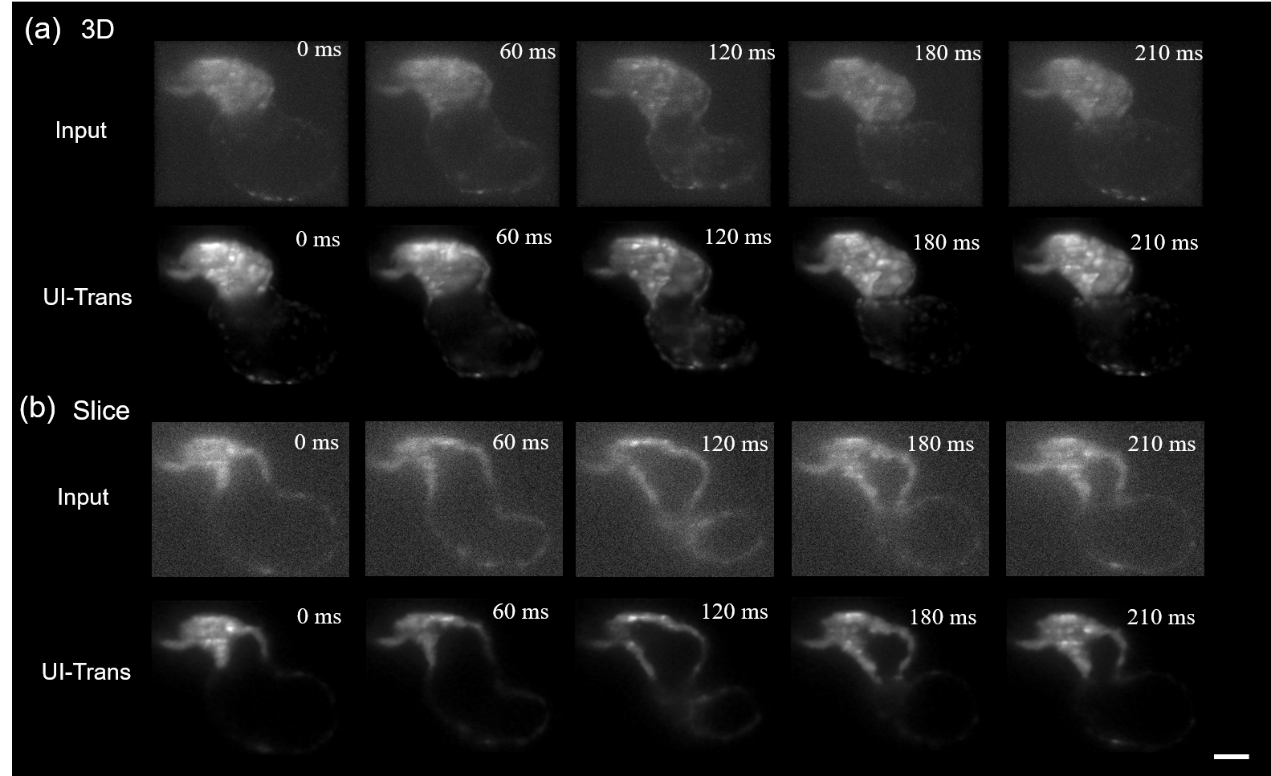


Fig. S20. *In vivo* cardiac dynamics investigation at 60 h.p.f. using UI-Trans-enhanced LSFM imaging. (a) The 3D visualizations of enhanced cardiac dynamics at embryonic stage (i.e. 60 h.p.f.) (b) The corresponding 2D slice images in x-y plane. Scale bar: 50 μm.

**Fig. S21. Restoration results using CNNs networks.**


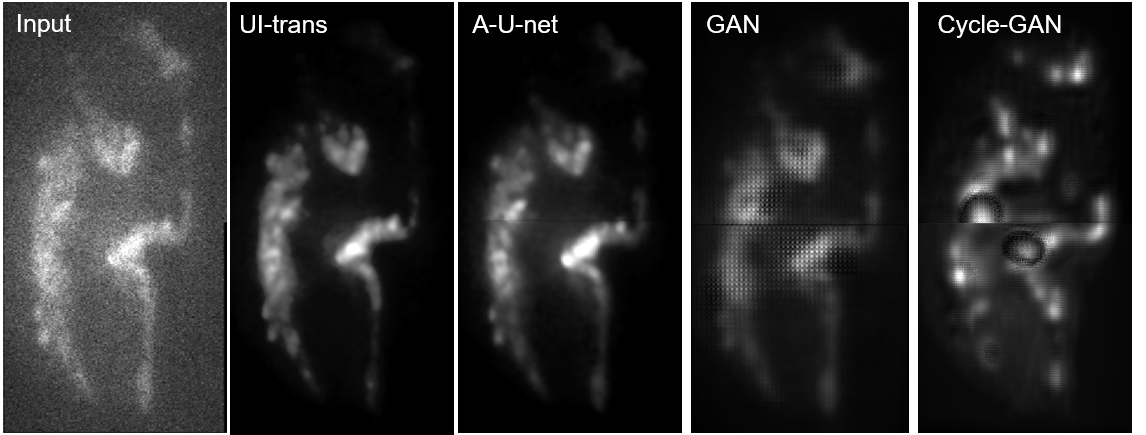


Fig. S21. Restoration results using CNNs networks. A-U-net: Attention U-net [6]; GAN: Generative Adversarial Networks[7]; Cycle-GAN: Cycle-Consistent Adversarial Networks[8].

Common CNN architectures were also employed to evaluate the restoration effectiveness of low-quality LSFM images. As shown in Fig. S21, the results demonstrate that adversarial networks such as GAN and Cycle-GAN, which previously excelled in 2D microscopic image restoration [9], suffer from severe image distortions and artifacts. The likely explanation could be the emphasis of adversarial networks on the consistency of image style at the expense of content accuracy. The addition of a gated attention mechanism in the Attention U-net shows an improved performance in this image restoration task, yet it remains less effective at suppressing scattering, especially compared to UI-Trans.

**S22. Experiment settings of *in vivo* zebrafish heartbeat imaging and training datasets.**

This work focused on presenting the enhancement of LSFM for *in vivo* imaging of zebrafish heart beating through the developed UI-Trans, and therefore the designed experiments and the corresponding results were specific to the conditions of zebrafish heart beating. In zebrafish heartbeat imaging, considering the continuous motion issue, the camera exposure time is usually limited to less than 5 ms to obtain clear LSFM slice images [10]. At this short exposure, in order to obtain sufficiently higher SNR images for biological investigations, it is common to increase the excitation power. However, it is noted that high illumination power could induce a cessation of zebrafish heartbeats, as observed in our sample (Tg (myl7: GFP)) under 0.3 mW illumination. Consequently, during *in vivo* imaging of zebrafish heartbeat, the illumination power was maintained at a relatively safe level (i.e. 0.1 mW), with an exposure time of 3 ms per slice. Under these conditions, the SNR of acquired images were extremely low, underscoring the demand for image restoration in the context of *in vivo* study of 3D cardiac dynamics of the model organism zebrafish.

To improve the restoration performance on *in vivo* heartbeat images, it is essential to match the degradation of the input image in training datasets (i.e. on *ex vivo* heart imaging) with *in vivo* heartbeat images. Therefore, during the acquisition of input images for the training dataset (i.e. on *ex vivo* samples), the same low illumination power as used for *in vivo* imaging (0.1 mW) was employed and the exposure time was increased to 10 ms to counteract the reduction in fluorescence intensity resulting from the fixation and optical clearing processes. During the LS-LSFM imaging for the acquisition of GT data, the exposure time was set at 1 ms per line due to the standard step size in this rolling shutter mode, which is crucial to maintain alignment between the input and GT images. Consequently, the excitation laser intensity was elevated to 10 mW to ensure that the dynamic range of the GT image aligns with the optimal specifications (>40000 a.u., 16 bits).

Reference

1. Rehman, A. et al. Convolutional Neural Network Transformer (CNNT) for Fluorescence Microscopy image Denoising with Improved Generalization and Fast Adaptation. *ArXiv.* (2024).

2. Tian, C. W. et al. A cross Transformer for image denoising. *Information Fusion.* **102**(2024).

3. Wang, Z. Y., Xie, Y. C. & Ji, S. W. Global voxel transformer networks for augmented microscopy. *Nat. Mach. Intell.* **3**, 161-171 (2021).

4. Wei, K. X. et al., "A Physics-based Noise Formation Model for Extreme Low-light Raw Denoising," in *2020 IEEE/CVF CONFERENCE ON COMPUTER VISION AND PATTERN RECOGNITION (CVPR),*  (2020), pp. 2755-2764.

5. Huang, F. et al. Video-rate nanoscopy using sCMOS camera-specific single-molecule localization algorithms. *Nature Methods.* **10**, 653-+ (2013).

6. Oktay, O. et al. Attention U-Net: Learning Where to Look for the Pancreas. Preprint at <https://arxiv.org/abs/1804.03999> (2018)

7. Isola, P. et al., Image-to-Image Translation with Conditional Adversarial Networks, in *2017 IEEE Conference on Computer Vision and Pattern Recognition (CVPR)*, 2017), 5967-5976.

8. Zhu, J. Y. et al., Unpaired Image-to-Image Translation Using Cycle-Consistent Adversarial Networks, in *2017 IEEE International Conference on Computer Vision (ICCV)*, 2017), 2242-2251.

9. Zhao, Y. et al. Isotropic super-resolution light-sheet microscopy of dynamic intracellular structures at subsecond timescales. *Nature Methods.* **19**, 359-369 (2022).

10. Mickoleit, M. et al. High-resolution reconstruction of the beating zebrafish heart. *Nat. Methods.* **11**, 919-922 (2014).
