# Supplementary figures and images for: Deep learning enhanced light sheet fluorescence microscopy for in vivo 4D imaging of zebrafish heart beating
Source: Light Sci Appl. 2025 Feb 25;14:92. doi: 10.1038/s41377-024-01710-z (PMC11850918; doi:10.1038/s41377-024-01710-z)

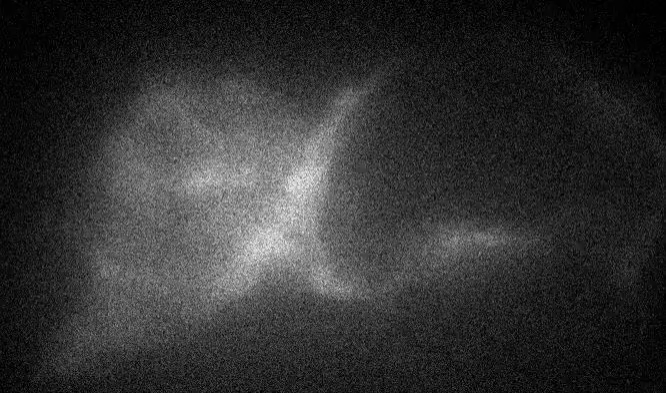

Supplement: Supplementary file 2 — movie S1 [file 41377_2024_1710_MOESM2_ESM.gif]

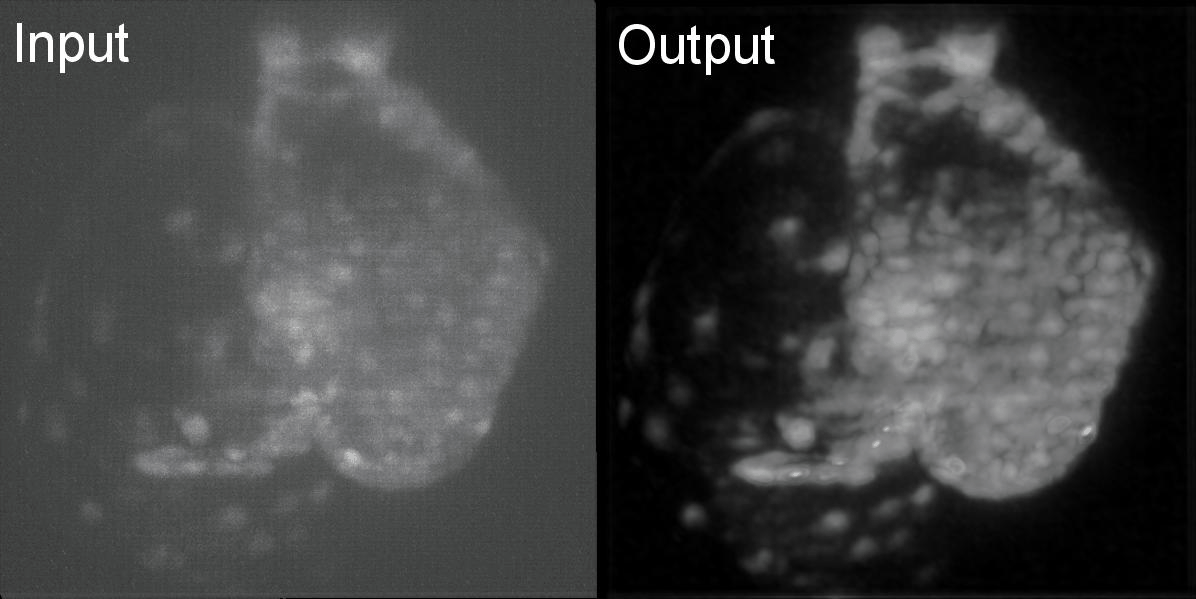

Supplement: Supplementary file 3 — movie S2 [file 41377_2024_1710_MOESM3_ESM.gif]

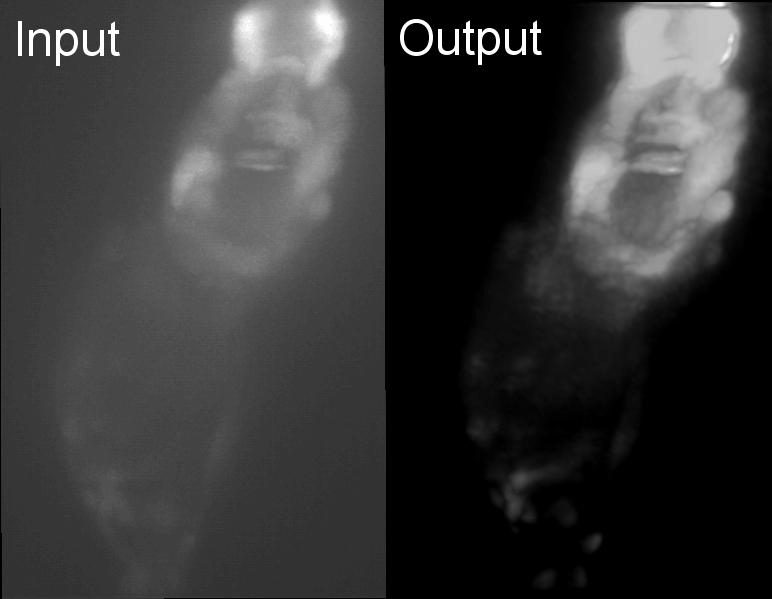

Supplement: Supplementary file 4 — movie S3 [file 41377_2024_1710_MOESM4_ESM.gif]

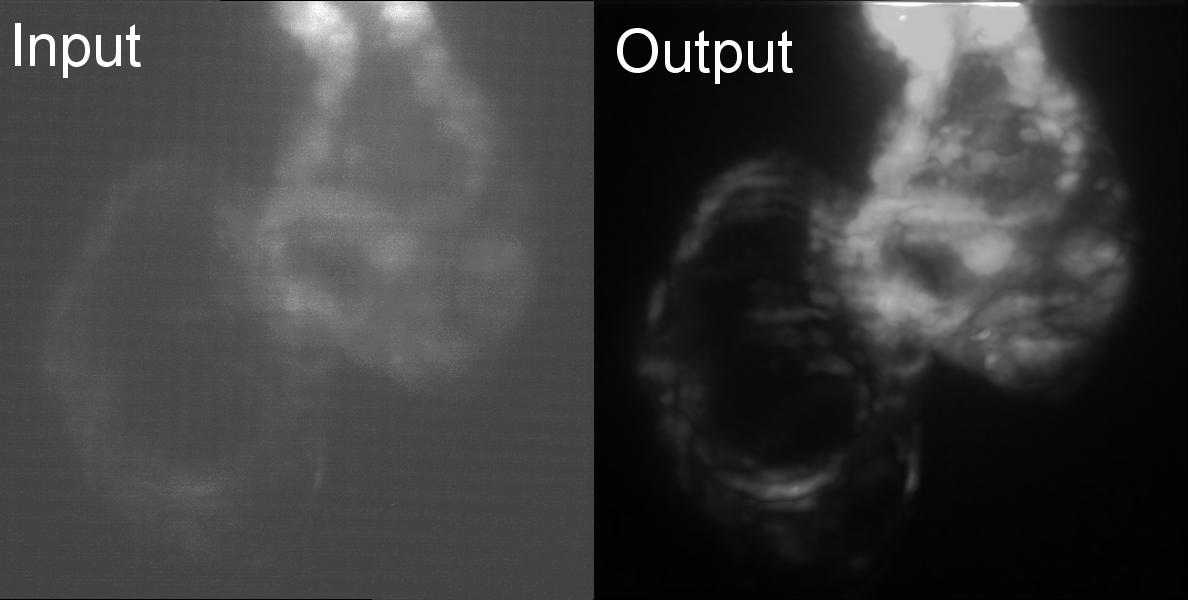

Supplement: Supplementary file 5 — movie S4 [file 41377_2024_1710_MOESM5_ESM.gif]
